# Construction of Heterometallic Coordination Nanosheets Comprising Both Inert and Labile Metal Ions Together via Metalloligand Approach

**DOI:** 10.1021/acs.inorgchem.5c00224

**Published:** 2025-04-03

**Authors:** Manas K. Bera, Sanjib Sarmah, Atanu Maity, Masayoshi Higuchi

**Affiliations:** † Polymers and Functional Materials Department, CSIR-Indian Institute of Chemical Technology (CSIR-IICT), Hyderabad 500007, India; ‡ Electronic Functional Macromolecules Group, Research Center for Macromolecules and Biomaterials, National Institute for Materials Science (NIMS), 1-1 Namiki, Tsukuba, Ibaraki 305-0044, Japan; § Department of Bioscience and Biotechnology, Indian Institute of Technology (IIT) Kharagpur, Kharagpur, West Bengal 721302, India; ∥ Academy of Scientific and Innovative Research (AcSIR), Ghaziabad 201002, India

## Abstract

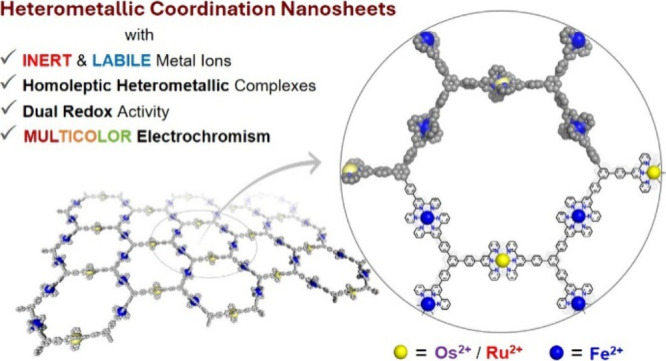

Construction of coordination nanosheets (CONASHs) containing both inert and labile metal ions together is fundamentally significant but remains synthetically unachievable until now and difficult to realize via conventional synthetic approach of CONASHs due to variable complexation conditions between heterometal ions and organic ligands. Here, we demonstrate a strategy to harness both inert and labile metal ions together into a CONASHs structure by introducing the concept of a metalloligand. Metalloligands comprising inert metal ion (Os^2+^/Ru^2+^) and free coordinating sites are designed and synthesized and can be further used as coordinating ligand to bind labile heterometal ion (Fe^2+^) for building of heterometallic CONASHs (HMCONASHs). Following this, two HMCONASHs containing homoleptic heterometallic complexes are constructed that show broad absorption and electrochemical window with reversible dual redox activity. Further, HMCONASH films exhibit multicolor electrochromism at different voltages, indicating their potential for various applications. This synthetic approach may open a window to create CONASHs with diverse structures and functions that are hard to achieve via a traditional synthetic approach.

## Abstract 2

We demonstrate a strategy to harness both inert and labile metal ions together into a CONASH structure by introducing the concept of a metalloligand.

Coordination nanosheets (CONASHs) are a class of 2D polymer materials that are conventionally prepared via bottom-up complexation of organic ligands and metal ions.^1−7^ The advantage of bottom-up CONASHs is that these can be constructed as free-standing thin films via interface-assisted synthesis, which offers building sheet structure from monolayer to multilayers with desired thickness and lateral dimensions.^3,8^ Additionally, the in situ film can be transferred on various substrates. Moreover, the structure and properties of CONASHs can be tuned by accessing different ligands and metal ions. In the past decade, various structural bottom-up CONASHs have been developed, and subsequently, their applications have been explored in different research directions.^9−17^

Although broad varieties of metal complex based CONASHs have been developed so far, all of them are based on only labile metal ions that typically form complexes at room temperature (25 °C), such as Fe, Co, Zn, Ni, Cu, Pb and Cd.^4,8,18−22^ Further, earlier reported CONASHs are mainly homometallic in nature except; one recently reported example of heterometallic CONASHs (HMCONASHs) was again constructed using a mixture of two labile metal ions (Ni and Cu) via one-pot synthesis.^23^ Schlüter et al. demonstrated partial transmetalation of the monolayer homosheet containing Zn^2+^ with Fe^2+^/Co^2+^/Pb^2+^ to make a heterosheet, where also only labile metal ions were studied.^5^

In contrast to labile metal ions, inert metal ions (such as Ru/Os) do not undergo reversible complexation with ligands at room temperature. Rather, they always form irreversible complexes with ligands at high temperature (>150 °C), and they mainly produce an insoluble mass of 3D coordination polymers instead of CONASHs.^24−26^ Therefore, it is a big challenge to introduce inert metal ions into a CONASH structure. An example of a 2D monolayer heterometallic nanosheet based on metal organic framework containing inert (Pt^2+^) and labile (Fe^2+^) metal ions was reported recently, where the nanosheet was mainly obtained as crystal via top-down method.^27^ Here inert metal ions mean the high binding strength of the metal ion with a ligand, which does not usually undergo substitution reaction at room temperature. Studies on the relative binding strength of terpyridine-based metal complexes revealed that Ru and Os show higher binding strengths than that of Fe for terpyridine-M^2+^-terpyridine type connectivity (where, M = Ru/Os/Fe).^24,25,28,29^

To overcome the above challenge associated with inert metal ions, here we showcase a novel strategy to incorporate an inert metal ion into CONASHs by introducing the concept of the metalloligand approach (Figure 1). The HMCONASHs containing both inert (Os^2+^/Ru^2+^) and labile (Fe^2+^) metal ions are created, where heterometal ions are in homoleptic bis(terpyridine)-metal complex environments. As terpyridine (2,2′:6′,2″-terpyridine) forms complexes with various type of inert and/or labile metal ions via terpyridine-M^2+^-terpyridine connectivity, it becomes a major choice as the binding motif for construction of CONASHs.^4,30−34^

**Figure 1:**
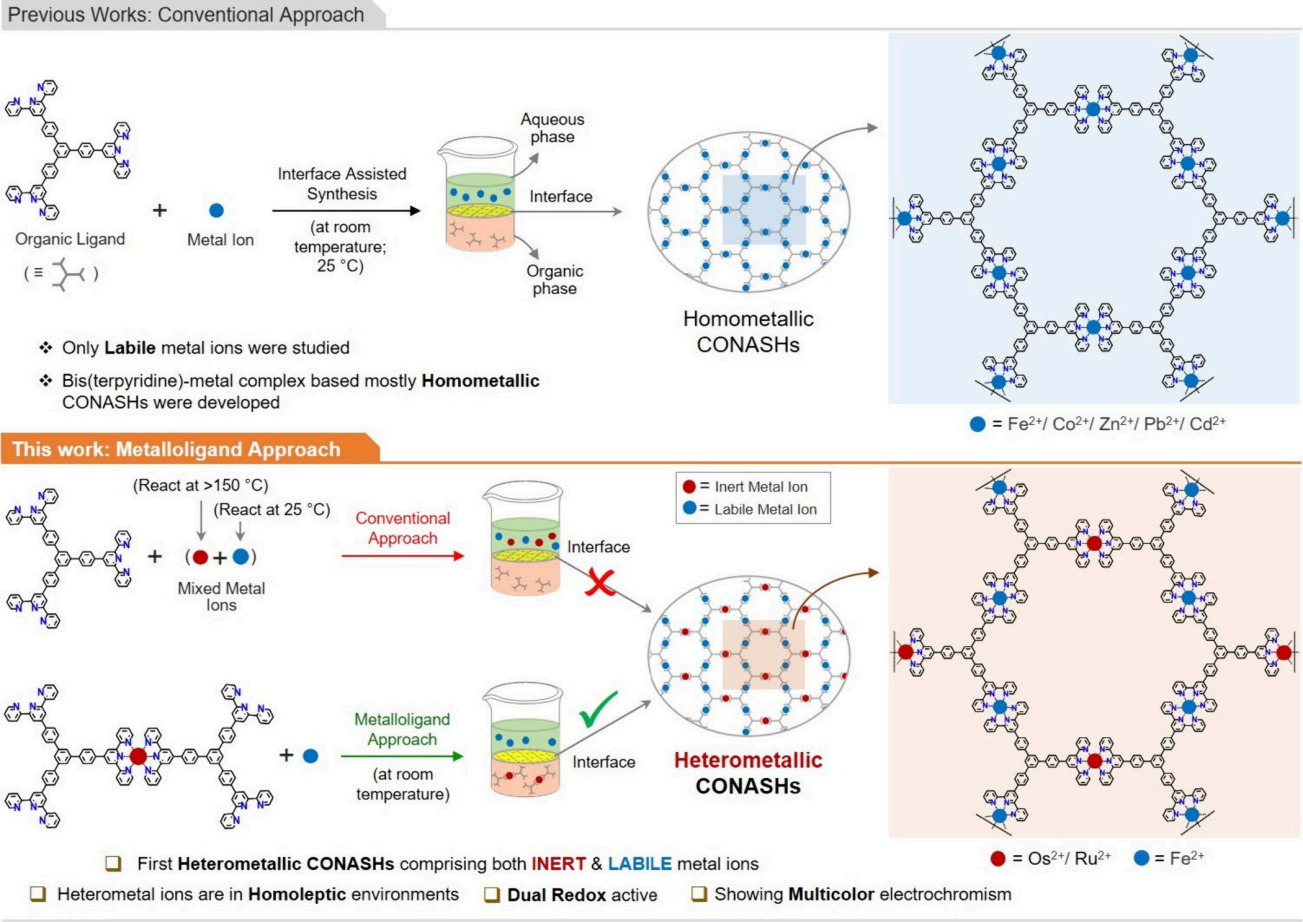
Construction of heterometallic coordination nanosheets (HMCONASHs); conventional approach vs our approach.

Conventionally, multilayered CONASHs are grown by interfacial (organic/aqueous) complexation at room temperature, where ligands are taken in the organic phase and metal ions in the aqueous phase (Figure 1; top).^8^ The HMCONASHs can be constructed via similar ways by taking a mixture of heterometal ions in the aqueous phase, but the process is limited to only labile metal ions.^23^ If a mixture of both inert and labile metal ions is used in the aqueous phase, the HMCONASHs are not obtained, as inert metal ions do not usually form metal complexes at room temperature. Therefore, we have designed and synthesized terpyridine based metalloligands (5Os and 5Ru) which contain inert metal ion (Os^2+^ or Ru^2+^) and four free terpyridine units that can further bind labile heterometal ions (Fe^2+^) via a conventional approach to build HMCONASHs (Figure 1; bottom).

The chemical structure and synthetic route for metalloligands are shown in Figure 2a (see the experimental section in the Supporting Information for details; Figures S1–S21). Starting from compound 1, a terpyridine based ligand 2 was synthesized. Then, intermediate complexes (3Ru and 3Os) of inert metal ions were obtained via complexation of ligand 2 and Os^2+^/Ru^2+^ at high temperature (≥180 °C), which do not usually undergo substitution reaction at room temperature. Finally, Suzuki coupling of 3Os/3Ru with compound 4 afforded the designed metalloligands (5Os and 5Ru).

**Figure 2:**
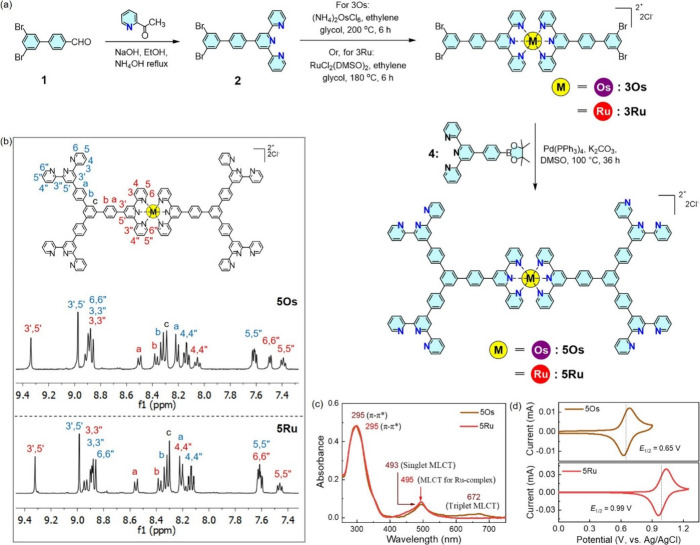
(a) Synthesis and chemical structure, (b) partial ^1^H NMR spectra, (c) UV–vis spectra, and (d) cyclic voltammograms of the metalloligands (5Os and 5Ru).

Figure 2b shows partial ^1^H NMR spectra of metalloligands with individual peak assignments, based on ^1^H–^1^H COSY and NOESY NMR spectra (Figures S14 and S15 and S19 and S20). Distinct proton signals for free and complexed terpyridine are observed, and peak integral values are well matched with theoretical values, indicating high purity of metalloligands. The mass of metalloligands (detected by MALDI-TOF) also well matched theoretical values (Figures S16 and S21). Formation of metalloligands was further confirmed by FTIR, UV–vis, and CV analysis. FTIR spectra indicated the presence of both complexed and free terpyridine units (Figure S22). In UV–vis spectra, characteristic singlet and triplet MLCT absorption bands for bis(terpyridine)-Os^2+^ complex in 5Os were observed at 493 and 672 nm, respectively, whereas the characteristic MLCT absorption band for the bis(terpyridine)-Ru^2+^ complex in 5Ru was observed at 495 nm (Figure 2c).^25^ The CV study revealed the expected one electron reversible redox feature for Os^2+^/Os^3+^ and Ru^2+^/Ru^3+^ couples in 5Os and 5Ru, respectively (Figure 2d).^30^

Free terpyridine units of metalloligands may undergo spontaneous complexation with labile Fe^2+^ to build heterometallic network structures of HMCONASHs; termed as HMCNOsFe and HMCNRuFe. The HMCONASHs may have several possible structural isomers depending on the position of the heterometal ions. The chemical structure and molecular model of a possible structural isomer for HMCONASHs is shown in Figure 3a and chemical structures of some other possible isomers are given in Figure S23. The HMCONASHs were constructed via liquid–liquid interface reaction, where 5Os/5Ru was taken in the organic phase (CH_2_Cl_2_/CH_3_OH; 4:1) as the bottom layer and iron dichloride in the aqueous phase as the top layer (Figure 3b; see experimental section for details). Continuous consumption of metalloligands to build HMCONASHs can be visually monitored by observing the change of the organic phase from a color to a transparent state. After 8 h, the purple film of HMCNOsFe and the red film of HMCNRuFe were formed at the interface. The HMCONASH films are insoluble in any organic/aqueous medium, indicating a network structure as proposed in Figure 3a. The films were deposited on desired substrates for further studies.

**Figure 3:**
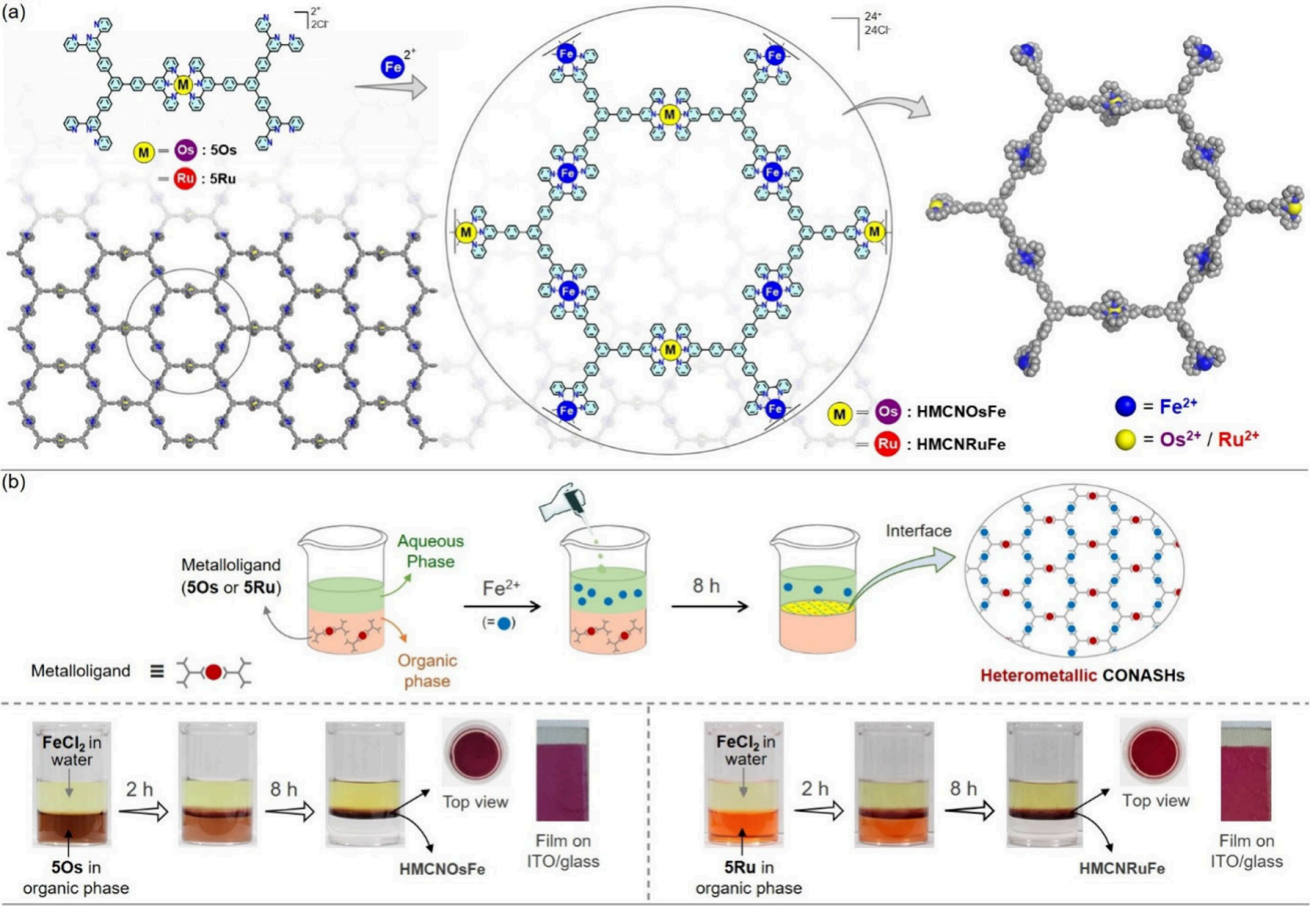
(a) Chemical structure and molecular model of a possible structural isomer for HMCONASHs (HMCNOsFe and HMCNRuFe). (b) Schematic view and photographs of liquid/liquid interfacial process used to synthesize HMCONASHs films (with deposited film on ITO/glass).

Optical and SEM studies of HMCONASHs revealed a flat, uniform, and sheet-like morphology (Figure 4a,b and Figure S24a,b). TEM images showed a layered structure (Figure 4c and Figure S24c), indicating layer-by-layer growth of nanosheets via a bottom-up process. AFM images also revealed a flat surface of HMCONASHs (Figure 4d and Figure S24d). From cross-sectional analysis of AFM images (Figure S25), thicknesses of HMCNOsFe and HMCNRuFe were found to be ∼380 and 440 nm, respectively. These thicknesses correspond to approximately 271 and 314 layers, respectively, for the HMCONASHs, considering the monolayer nanosheet thickness of 1.4 nm as proposed by Schlüter et al.^7^ However, the thickness of nanosheets can be varied by controlling reaction conditions.^3^ The constitutive elements were homogeneously distributed throughout the nanosheets as confirmed by EDX elemental mapping using TEM (Figure 4e and Figure S24e), where C, N, Os/Ru, and Fe form a nanosheet framework with Cl in the counteranion part. Further structural analysis of HMCONASHs by XPS indicated characteristic binding energy peaks at 399.2 707.9, 720.6, 52.9, 50.2, and 190 eV, for N 1s, Fe 2p, Os 4f, and Cl 2p core levels, respectively, for HMCNOsFe (Figure 4f–j). Similarly, for HMCNRuFe, the peaks at 399.2, 707.9 and 720.6, 280.2, and 190 eV, for N 1s, Fe 2p, Ru 3p, and Cl 2p core levels, were observed (Figure S24f–j). From XPS analysis, the atomic ratio of mixed metal to N was calculated to be 1:6.2 [(Os+Fe):N] in HMCNOsFe and 1:6.1 [(Ru+Fe):N] in HMCNRuFe, which conform to the ideal value of mixed metal to N ratio (1:6). Moreover, the ratio of heterometal ions (Os/Ru:Fe) was determined to be 1:2.04 and 1:2.07 for HMCNOsFe and HMCNRuFe, respectively, which are also close to an ideal ratio of 1:2.

**Figure 4:**
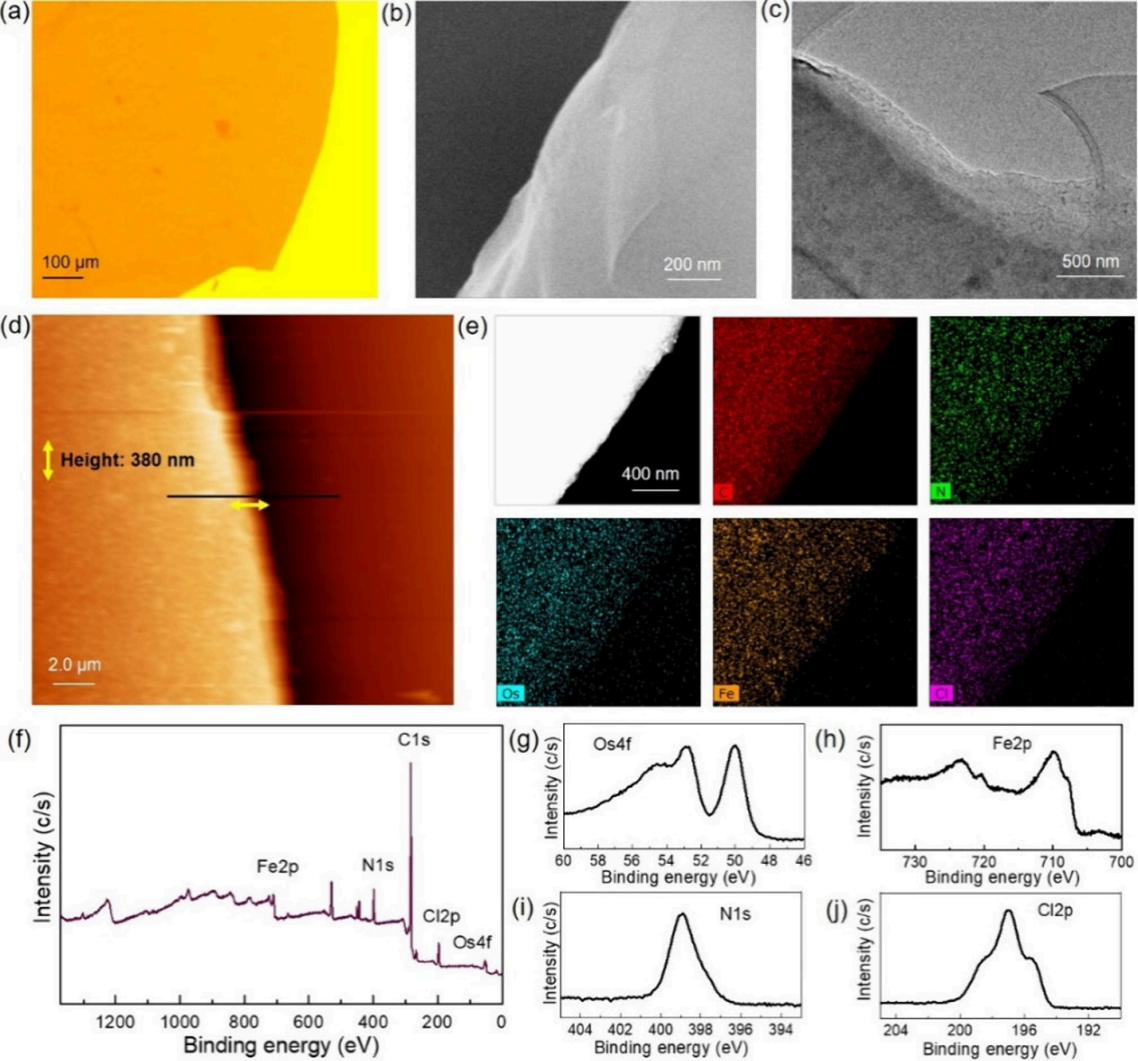
Characterization of HMCNOsFe film. (a) OM, (b) SEM, (c) TEM, (d) AFM images, (e) TEM/EDX elemental mapping, (f) broad and (g--j) narrow scan XP spectra focusing of Os 4f, Fe 2p, N 1s, and Cl 2p core levels, respectively.

FTIR spectra of HMCONASHs revealed the presence of C=C stretching frequencies for complexed terpyridine units only in comparison to metalloligands, which is a common observation for terpyridine-metal complexes (Figure S26).^3^ The PXRD profile showed a broad peak at higher 2θ values of 22.90° and 23.60° for HMCNOsFe and HMCNRuFe, indicating π-π stacking between consecutive layers of nanosheets with interlayer distances of ∼3.90 and ∼3.80 Å, respectively (Figure S27a,b). TGA analysis indicated excellent thermal stabilities (up to 580 °C with <20% weight loss) of HMCONASH films, reflecting the robustness of coordination bonds in the nanosheets (Figure S27c).

The HMCONASHs displayed a broad absorption window due to the mixing of heterometallic complexes. For the HMCNOsFe film, the 350 nm peak represents aromatic π-π* transition, the 496 and 674 nm peaks are for singlet and triplet MLCT absorption of bis(terpyridine)-Os^2+^ complexes, and the 573 nm peak represents bis(terpyridine)-Fe^2+^ complexes (Figure S28a).^35^ Similarly, the HMCNRuFe film covers the π-π* transition band at 340 nm, the MLCT absorption of bis(terpyridine)-Ru^2+^ complexes at 496 nm, and the MLCT absorption of bis(terpyridine)-Fe^2+^ complexes at 574 nm (Figure S28b).^25^ The CV study revealed two reversible redox waves with *E*_1/2_ of 0.61 and 0.78 V for HMCNOsFe, which are assignable to Os^2+^/Os^3+^ and Fe^2+^/Fe^3+^ couples. For HMCNRuFe, reversible waves with *E*_1/2_ values of 0.79 and 0.92 V can be assigned to Fe^2+^/Fe^3+^ and Ru^2+^/Ru^3+^ couples (Figure 5a,b). The area under the curve for the two redox waves also reflects the ratio of inert and labile heterometal ions to be ∼1:2, which is consistent with the ratio obtained from XPS analysis.

**Figure 5:**
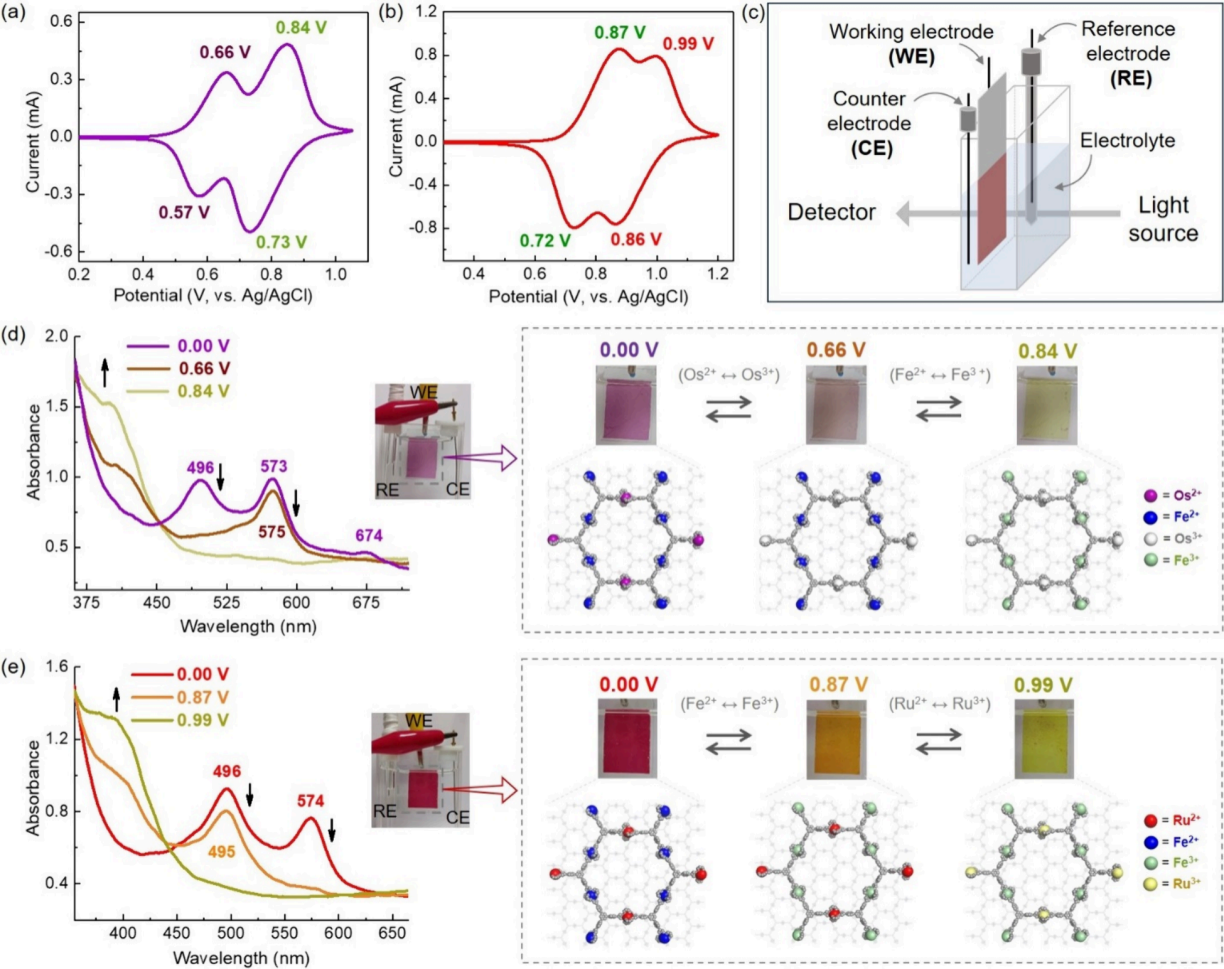
Cyclic voltammogram of (a) HMCNOsFe and (b) HMCNRuFe films. (c) Schematic view for in situ spectroelectrochemical measurement of nanosheets films. In situ UV–vis spectra at different potentials for (d) HMCNOsFe and (e) HMCNRuFe films. The inset shows photographs of film at different voltages and mechanistic view for redox-state change of heterometal ions in nanosheets structure.

During CV analysis, HMCONASH films (on ITO/glass) display an optical color change upon voltage alteration. So, in situ spectroelectrochemical measurement was conducted to explore their possible applications (Figure 5c). In forward bias, when the potential reached 0.66 V, the color of the HMCNOsFe film changed from purple to light magenta due to oxidation of Os^2+^ ions, which is also reflected by the disappearance of singlet and triplet MLCT absorption bands corresponding to bis(terpyridine)-Os^2+^ complexes. When the potential reached 0.84 V, the color of the film further changed to greenish-yellow due to oxidation of Fe^2+^ ions, and consequently, the MLCT absorption band corresponding to bis(terpyridine)-Fe^2+^ complexes vanished. In backward bias, the color of the film reversibly changed to its initial state in stepwise manner upon successive reduction of heterometal ions with the reappearance of corresponding absorption bands (Figure 5d). Similarly, the HMCNRuFe film changed its color from red to yellow (at 0.87 V due to oxidation of Fe^2+^ ions) and to greenish yellow (at 0.99 V due to oxidation of Ru^2+^ ions) in forward bias with stepwise disappearance of MLCT absorption bands of bis(terpyridine)-Fe^2+^ complexes and bis(terpyridine)-Ru^2+^ complexes. In backward bias, the color of the film and absorption bands successively appeared in a reversible manner (Figure 5e). This kind of dual redox triggered multicolor electrochromism of HMCONASH films demonstrates their potentiality for various applications including decorative displays, intelligent windows, and logic gates.^36−39^ Moreover, a broad optical and electrochemical window with dual redox activity of HMCONASHs may lead to interesting optical, electrochemical, and energy related applications.^40−46^

In summary, HMCONASHs containing inert and labile metal ions together were constructed by introducing the concept of the metalloligand approach, where heterometal ions reside in homoleptic coordination environments (see Table ST1 for comparison with earlier reports). Mixing of redox active heterometallic complexes into nanosheet structures not only results in a broad optical window and electrochemical window but also leads to dual redox triggered multicolor electrochromism, which may find potential optical, electrochemical, electrochromic, and energy storage applications. We believe that this work will open up new opportunities not only to enrich the family of CONASHs but also to create new functionalities by introducing heterometal ions of desired combinations that are previously inaccessible via conventional methods.

## Supporting Information Available

Experimental details; synthesis procedures; ^1^H, ^13^C, 2D COSY, and NOESY NMR spectra; MALDI-TOF mass spectra; IR and UV–vis spectra; AFM profiles; TGA; XRD and additional figures; table of comparison with earlier reports (PDF)
