# Supplementary material for: Construction of Heterometallic Coordination Nanosheets Comprising Both Inert and Labile Metal Ions Together via Metalloligand Approach
Source: Inorg Chem. 2025 Apr 3;64(18):8837–44. doi: 10.1021/acs.inorgchem.5c00224 (PMC12076548; doi:10.1021/acs.inorgchem.5c00224)
Supplement: Supplementary file 1 — ic5c00224_si_001.pdf [file ic5c00224_si_001.pdf]

Supporting Information

for

Construction of Heterometallic Coordination Nanosheets Comprising Both Inert and Labile Metal Ions Together via Metalloligand Approach

Manas K. Bera,[*,a,b,d] Sanjib Sarmah,[a,d] Atanu Maity,[c] and Masayoshi Higuchi*[*,b]*

[a] Polymers and Functional Materials Department, CSIR-Indian Institute of Chemical Technology (CSIR-IICT), Hyderabad 500007, India

[b] Electronic Functional Macromolecules Group, Research Center for Macromolecules and Biomaterials, National Institute for Materials Science (NIMS), 1-1 Namiki, Tsukuba, Ibaraki 305-0044, Japan

[c] Department of Bioscience and Biotechnology, Indian Institute of Technology (IIT) Kharagpur, West Bengal 721302, India

[d] Academy of Scientific and Innovative Research (AcSIR), Ghaziabad 201002, India

* mkb.bera@yahoo.com; manas.497@csiriict.in

* HIGUCHI.Masayoshi@nims.go.jp

Table of Contents

Contents **Page No**

1.	Experimental Section	S3
1.1.	Materials and general information	S3
1.2.	Instrumental characterizations and methods	S4
2.	Synthesis of intermediate compounds, metalloligands and heterometallic coordination nanosheets (HMCONASHs) (^{1}H, ^{13}C, 2D COSY, and NOESY NMR spectra, MALDI-TOF mass spectra; Figure S1-S21)	S5-S26
3.	Supplementary figures	
	Figure S22: FT-IR spectra of metalloligands and other compounds	S26
	Figure S23: Possible network structures of the HMCONASHs	S27
	Figure S24: Characterization of HMCNRuFe film	S28
	Figure S25: AFM height profile of HMCONASHs	S29
	Figure S26: FT-IR spectra of HMCONASHs	S30
	Figure S27: XRD and TGA of HMCONASHs	S31
	Figure S28: UV-vis spectra of HMCONASHs	S32
4.	Table ST1: Comparison of our work with earlier reports	S33-S34
5.	References	S35

1. Experimental Section

1.1. Materials and general information

The chemicals were purchased from Tokyo Chemical Industry (TCI, India) Co. Ltd. and Sigma-Aldrich. Spectroscopic and dry grade solvents were purchased from Finar Chemicals, AVRA, and Spectrochem Pvt. Ltd. Chemical and solvents were used without purification. Indium tin oxide coated glass substrate (ITO/glass) was purchased from Sigma-Aldrich. The ITO/glass substrates were washed with ethanol and acetone before use. The reactions were monitored by thin layer chromatography (TLC) under 254 and 365 nm UV light. Column chromatography was done using aluminium oxide basic (activity I-II according to brockman) purchased from Finar. Compounds 1 was synthesized according to reported literature procedure.[1]

1.2. Instrumental Characterizations and methods

Nuclear Magnetic Resonance (NMR) Spectroscopy

The ^{1}H, ^{13}C, 2D COSY, and NOESY NMR spectra were recorded on Bruker spectrometer (400 MHz, 400 Avance NMR spectrometer running X-WIN NMR software), using $CDCl_3$, and mixture of CD_2Cl_2/CD_3OD as solvents. The chemical shifts are expressed in parts per million (ppm) relative to tetramethylsilane (TMS) standard. Signal multiplicities are expressed as follow: s for singlet; d for doublet; t for triplet; m for multiplet, dd for doublet of doublet, and dt for doublet of triplet.

Matrix assisted laser desorption ionization-time of flight mass (MALDI-TOF) spectrometry

Mass of intermediate compounds and metalloligands were measured using MALDI-TOF mass spectrometer (Shimadzu Biotech Axima Performance 2.9.3.20110624) using 1,8,9-trihydroxyanthracene as a matrix.

Fourier transform infrared (FT-IR) spectroscopy

The FT-IR measurements were conducted on Perkin-Elmer Spectrum-100 FTIR spectrometer at a frequency range from 4000 cm^{-1} to 400 cm^{-1} using KBr pellets.

Optical microscopy (OM)

The OM images of heterometallic CONASHs films were captured using OLYMPUS-CKX41 microscopy. The films of heterometallic CONASHs deposited on glass substrate were used for the study.

Field Emission Scanning electron microscopy (FE-SEM)

The SEM analysis was performed on Field Emission Scanning Electron Microscope (S8000, Hitachi) operated at 10kV after sputter-coating with a platinum coater (E-1030 ion sputter, Hitachi). The nanosheets were dispersed in CH_2Cl_2/ethanol (1:1) mixed solvent and drop casted on silicon wafers that were used for FE-SEM study.

Transmission electron microscopy

The TEM analysis was carried out on a FEI Talos F200X HR-TEM operating at 200 kV. The samples for TEM study were prepared by dispersing nanosheets in CH_2Cl_2/ethanol (1:1) mixed solvent and drop casted on 150-mesh carbon coated copper grid followed by drying under vacuum. Energy-dispersive X-ray (EDX) analysis was further performed on TEM to get elemental mapping.

Atomic force microscopy (AFM)

The AFM images were captured in Bruker Innova-S2 instrument in tapping mode. Samples were prepared by dispersing nanosheets in CH_2Cl_2/ethanol (1:1) mixed solvent followed by spin coating on freshly cleaned mica surface.

XPS

The XPS analysis was performed on PHI Quantera SXM (ULVAC-PHI) instrument using X-ray (Al Kα mono) 1.4 × 0.1mm 100 W (20 kV, 5 mA), take off angle: 45deg, survey spectra: pass energy 280 eV, energy step 0.5 eV. Energy calibration; C1s peak as 285.0 eV.

Powder X-ray diffraction (PXRD)

The PXRD was measured using a Bruker D8 Advance Davinci diffractometer in Bragg-Brentano (reflection) geometry. The instrument was equipped with a Cu Kα X-ray source (wavelength of 1.5418 Å. The X-rays were generated at 40 kV voltage and 30 mA current, and scanned the range of 2-theta from 2° to 80°.

UV-vis spectroscopy

The UV-visible absorption spectra were recorded in Shimadzu (UV-2550) UV-visible spectrophotometer taking nanosheets films on ITO/glass substrate.

Electrochemical study

Electrochemical and spectroelectrochemical measurements were done on CH-Instrument in a three-electrode system, where nanosheets deposited ITO/glass substrate as working electrode, platinum wire as counter electrode, and Ag/AgCl as reference electrode, were used. An integrated Ocean Optics modular spectrometer connected with electrochemical analyser was used for in situ UV-vis absorption measurement of the heterometallic CONASHs on ITO/glass upon application of different voltages.

Molecular Modelling

Structural optimization was conducted in Avogadro molecular visualizer.[2] A total of ten thousand steps energy minimization were performed using conjugate gradient algorithm.[3] The UFF (Universal force field) force field was used to parameterize the system.[4]

2. Synthesis of intermediate compounds, metalloligands, and heterometallic coordination nanosheets (HMCONASHs)

2.1. Synthetic of compound 2

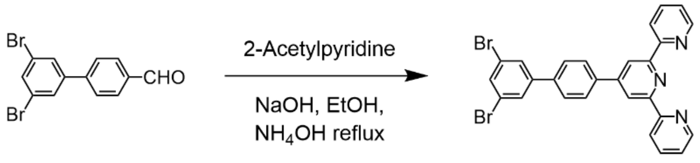

2-Acetylpyridine (87.2 mg, 0.72 mmol) was added to a solution of compound 1 (115.6 mg, 0.34 mmol) in ethanol (30 mL), and then NaOH (100.8 mg, 2.52 mmol) was added to it. After that the solution was stirred overnight at room temperature, $NH_3 \cdot H_2O$ (8 mL, 28-30 %) was added and the solution was heated to 34 °C for 24 h. The mixture was cooled, filtered and the solid was collected by filtration followed by washed with ice-cold ethanol (3 times) to get compound 2 as a white solid (116.3 mg, 63% yield). **^{1}H NMR** ($CDCl_3$, 400 MHz, ppm): δ 8.78 (s, 2H), 8.75-8.74 (m, 2H), 8.70-8.68 (m, 2H), 8.01 (d, 2H), 7.90 (dt, 2H), 7.73(d, 2H), 7.69-7.67 (m, 3H), 7.39 (dt, 2H).

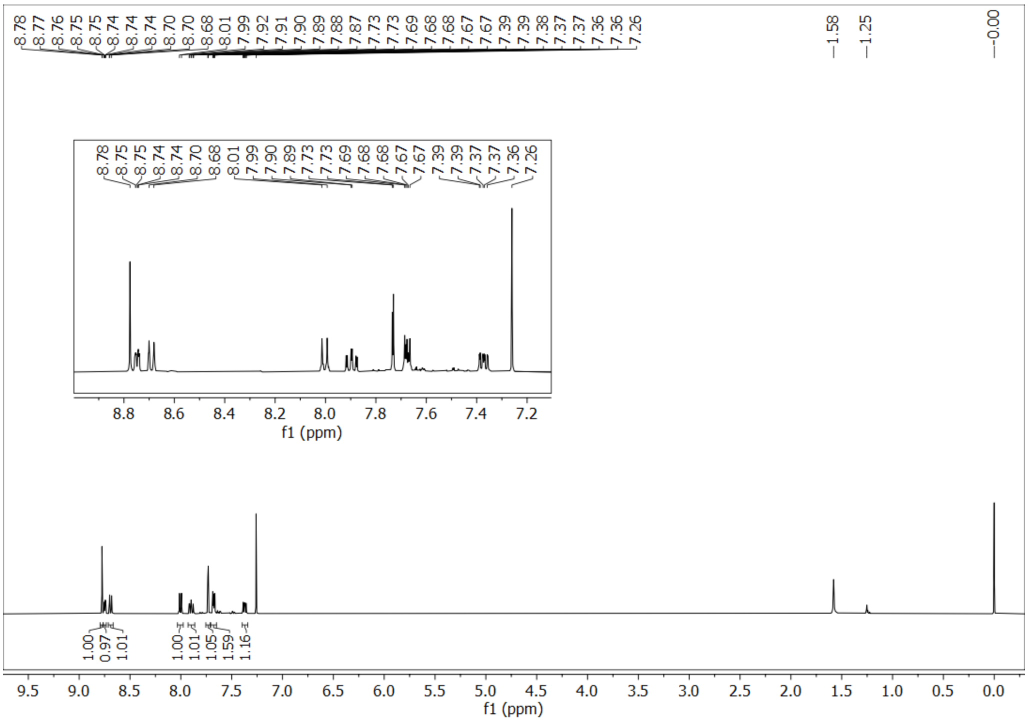

Figure S1. ^{1}H NMR spectrum of 2 in $CDCl_3$.

2.2. Synthesis of 3Os

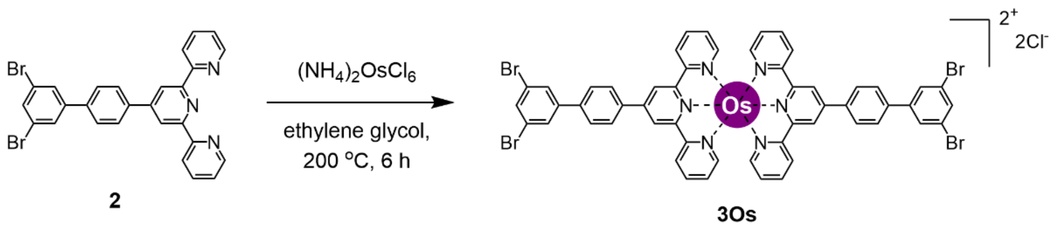

Compound 2 (234.7 mg, 0.43 mmol) and $(NH_4)_2OsCl_6$ (88 mg, 0.2 mmol) were stirred in 8 mL dry and degassed ethylene glycol under nitrogen atmosphere for 6 h at 200 °C. The reaction mixture was cooled and excess THF was added for getting precipitation. The precipitate was filtered off to get black powder which was purified by column chromatography (basic Al_2O_3) eluting with DCM/MeOH (first 10:1 and then 4:1) to isolate 3Os (218.3 mg, 81% yield). **^{1}H NMR** (CD_2Cl_2/CD_3OD; 1:1, 400 MHz, ppm): δ 9.37 (s, 4H), 8.96 (d, 4H), 8.45 (d, 4H), 8.10 (d, 4H), 8.02-7.98 (m, 8H), 7.90 (s, 2H), 7.47 (d, 4H), 7.36 (dd, 4H). **^{13}C NMR** (CD_2Cl_2/CD_3OD; 1:1, 400 MHz, ppm): δ 161.43, 156.68, 153.50, 148.96, 144.99, 141.90, 139.76, 137.36, 134.80, 130.55, 130.30, 129.65, 129.56, 126.72, 124.90, 121.73. **MALDI-TOF-MS** (*m/z*): 1274.08 $[3Os\text{-}2Cl^-]^{2+}$ (calculated *m/z* = 1273.92).

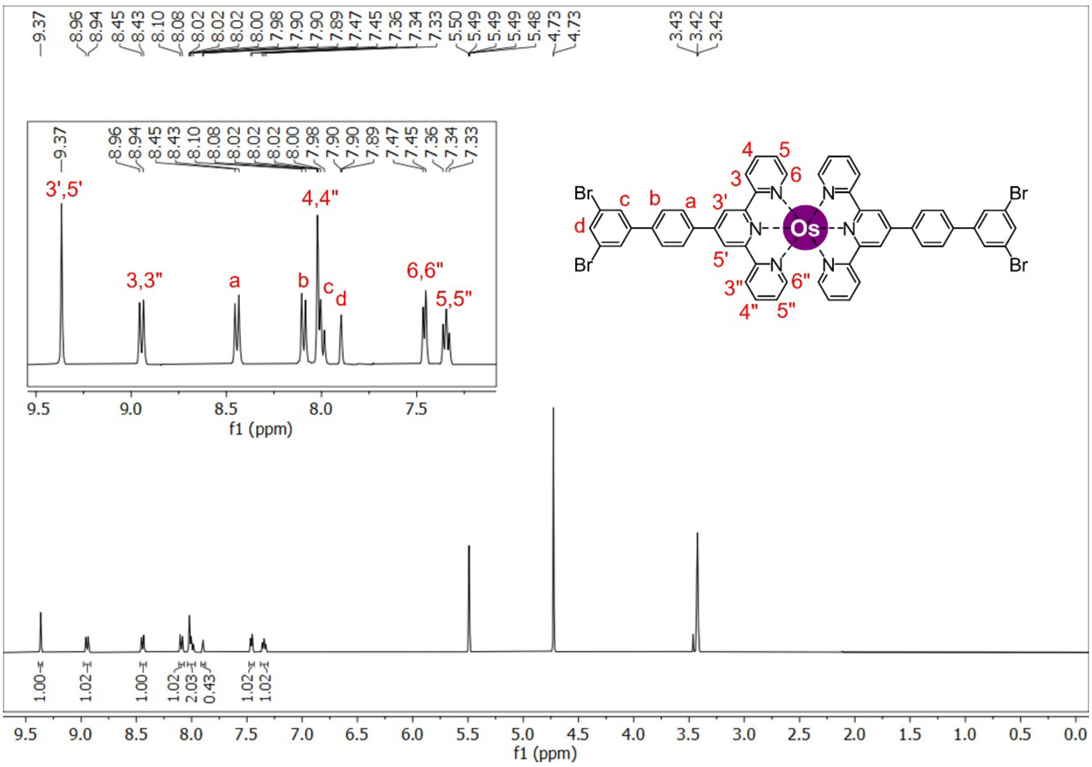

Figure S2. ^{1}H NMR spectrum of 3Os in CD_2Cl_2/CD_3OD; 1:1 (v/v).

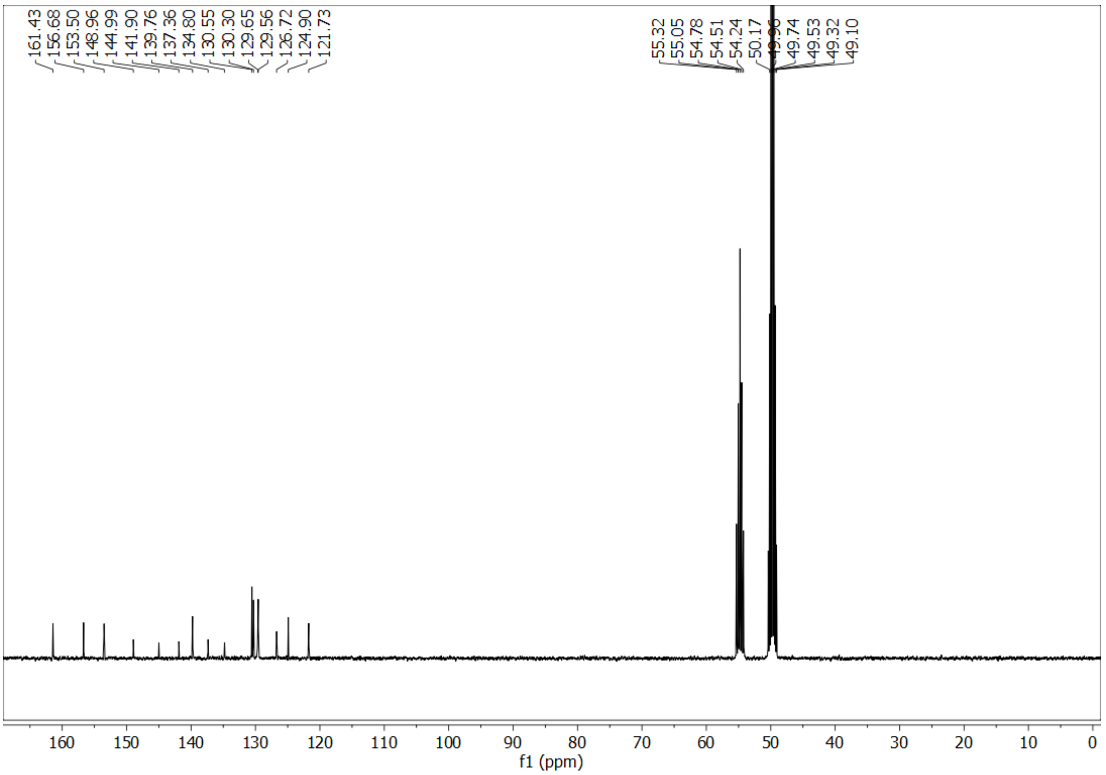

Figure S3. ^{13}C NMR spectrum of 3Os in CD_2Cl_2/CD_3OD; 1:1 (v/v).

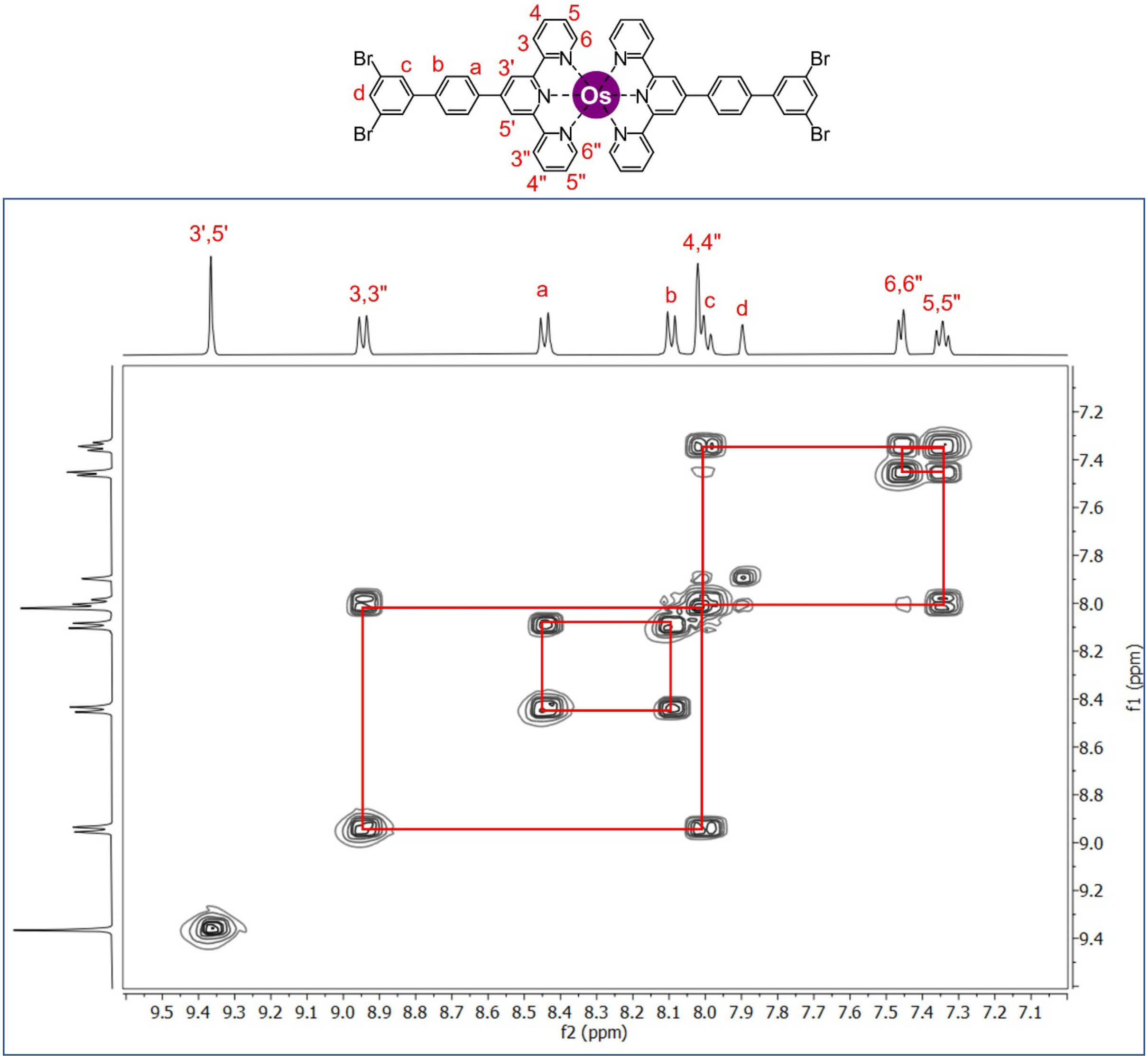

Figure S4. COSY NMR spectrum of 3Os in CD_2Cl_2/CD_3OD; 1:1 (v/v).

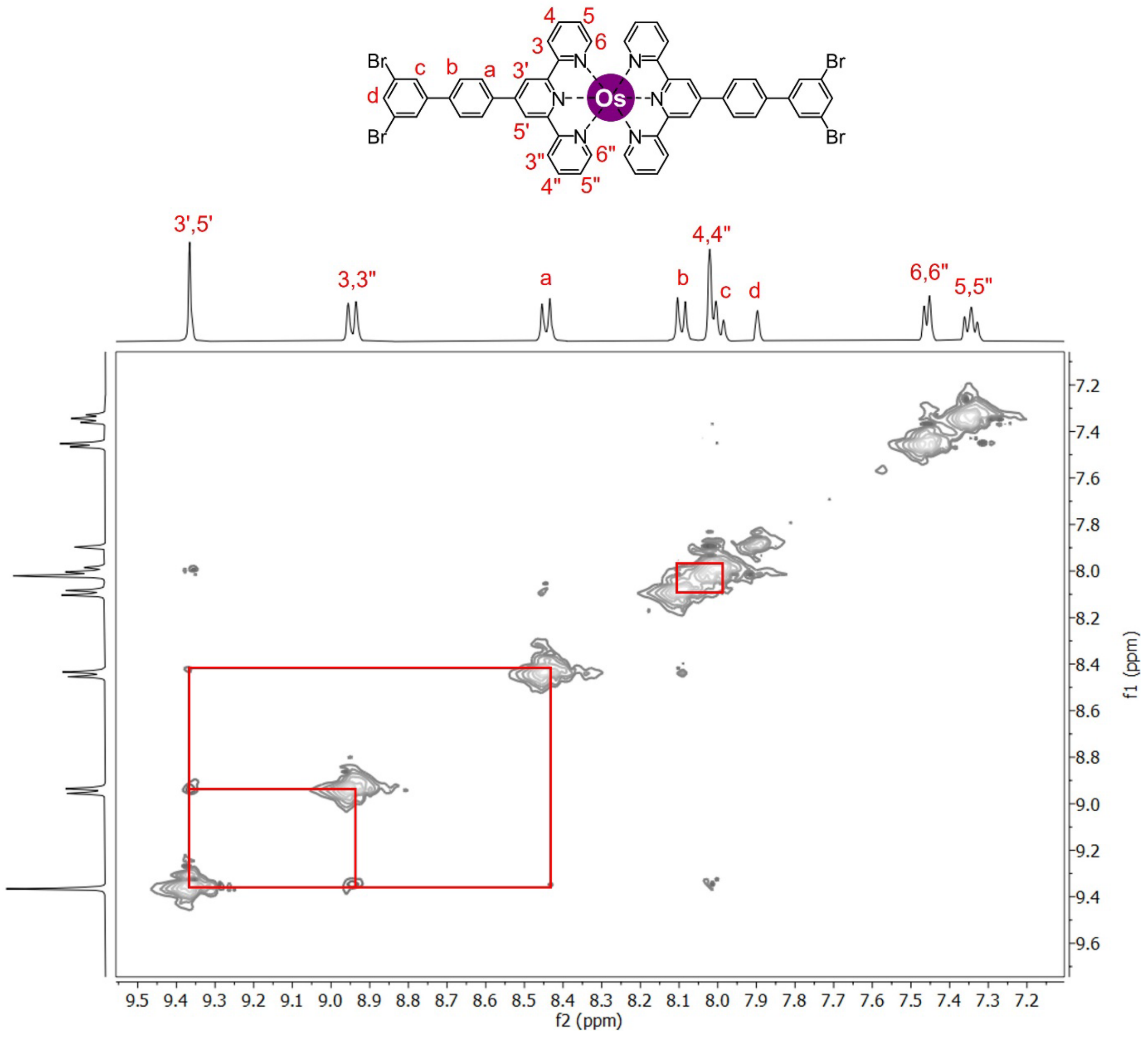

Figure S5. NOESY NMR spectrum of 3Os in CD_2Cl_2/CD_3OD; 1:1 (v/v).

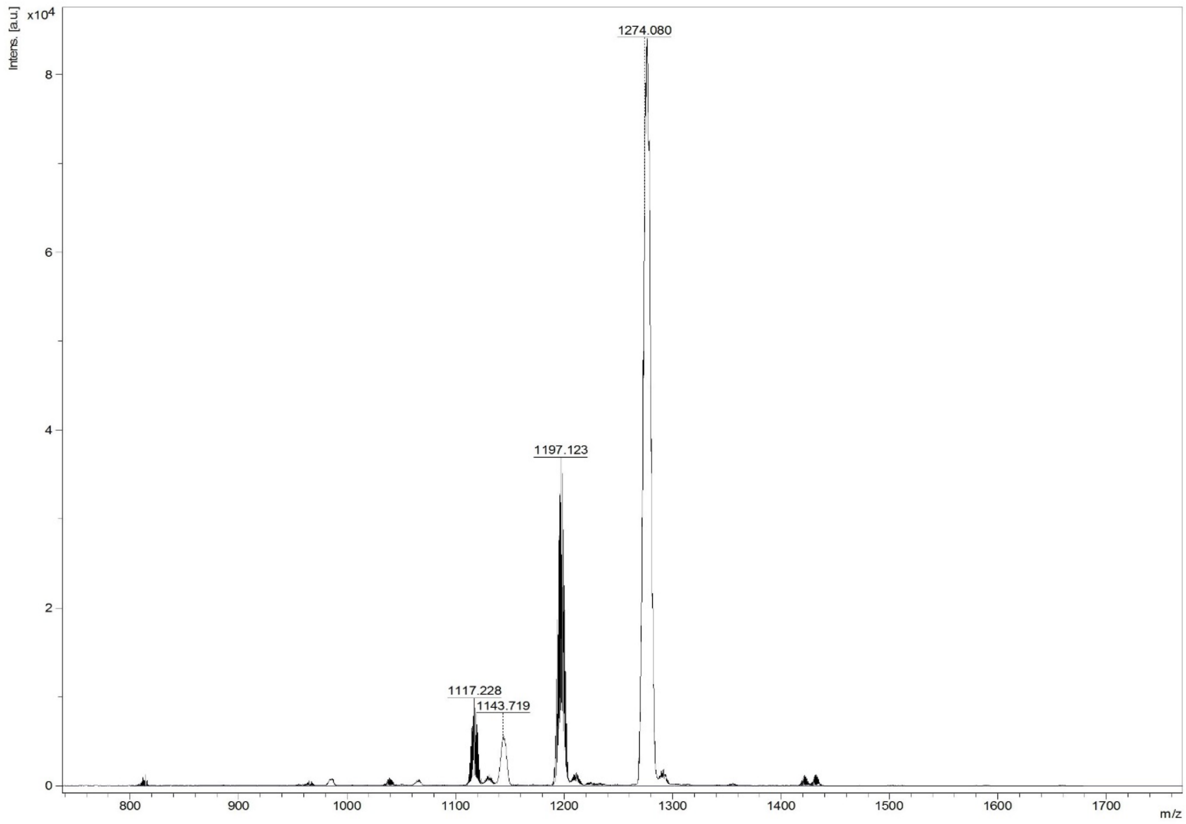

Figure S6. MALDI-TOF mass spectrum of 3Os.

2.3. Synthesis of 3Ru

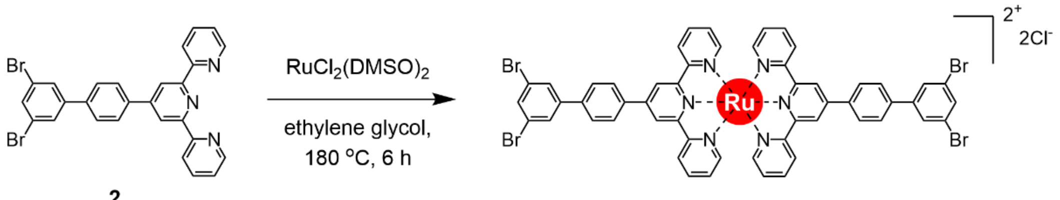

Compound 2 (200 mg, 0.37 mmol) and $RuCl_2(DMSO)_2$ (80 mg, 0.17 mmol) were stirred in 8 mL dry and degassed ethylene glycol under nitrogen atmosphere for 6 h at 180 °C. The reaction mixture was cooled and excess THF was added for getting precipitation. The precipitate was filtered off to get red powder which was purified by column chromatography (basic Al_2O_3) eluting with DCM/MeOH (first 10:1 and then 4:1) to isolate 3Ru (188.2 mg, 88% yield). **^{1}H NMR** (CD_2Cl_2/CD_3OD; 1:1, 400 MHz, ppm): δ 9.36 (s, 4H), 8.99 (d, 4H), 8.51 (d, 4H), 8.14-8.08 (m, 8H), 8.03 (s, 4H), 7.91 (s, 2H), 7.60 (d, 4H), 7.43-7.39 (m, 4H). **^{13}C NMR** (CD_2Cl_2/CD_3OD; 1:1, 400 MHz, ppm): δ 159.65, 157.07, 153.50, 149.82, 145.06, 141.89, 137.98, 134.83, 130.51, 129.97, 129.81, 129.45, 126.56, 124.95, 123.03. **MALDI-TOF-MS** (*m/z*): 1183.96 $[3Ru\text{-}2Cl^-]^{2+}$ (calculated *m/z* = 1183.86).

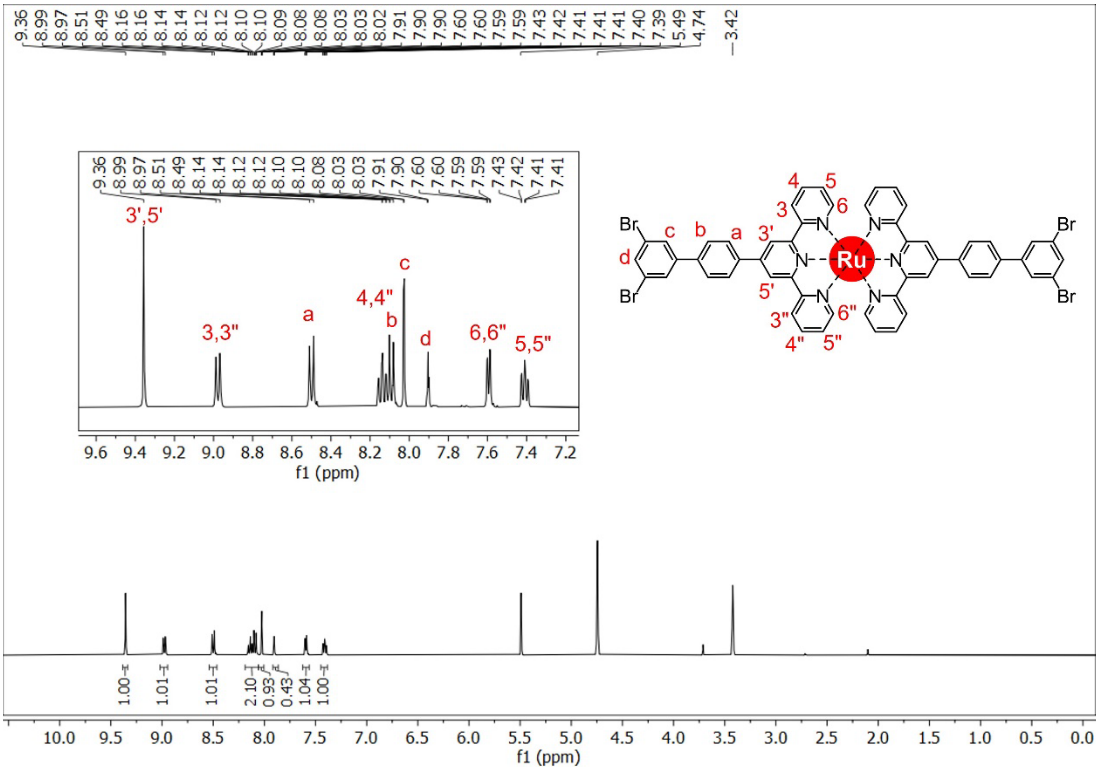

Figure S7. ^{1}H NMR spectrum of 3Ru in CD_2Cl_2/CD_3OD; 1:1 (v/v).

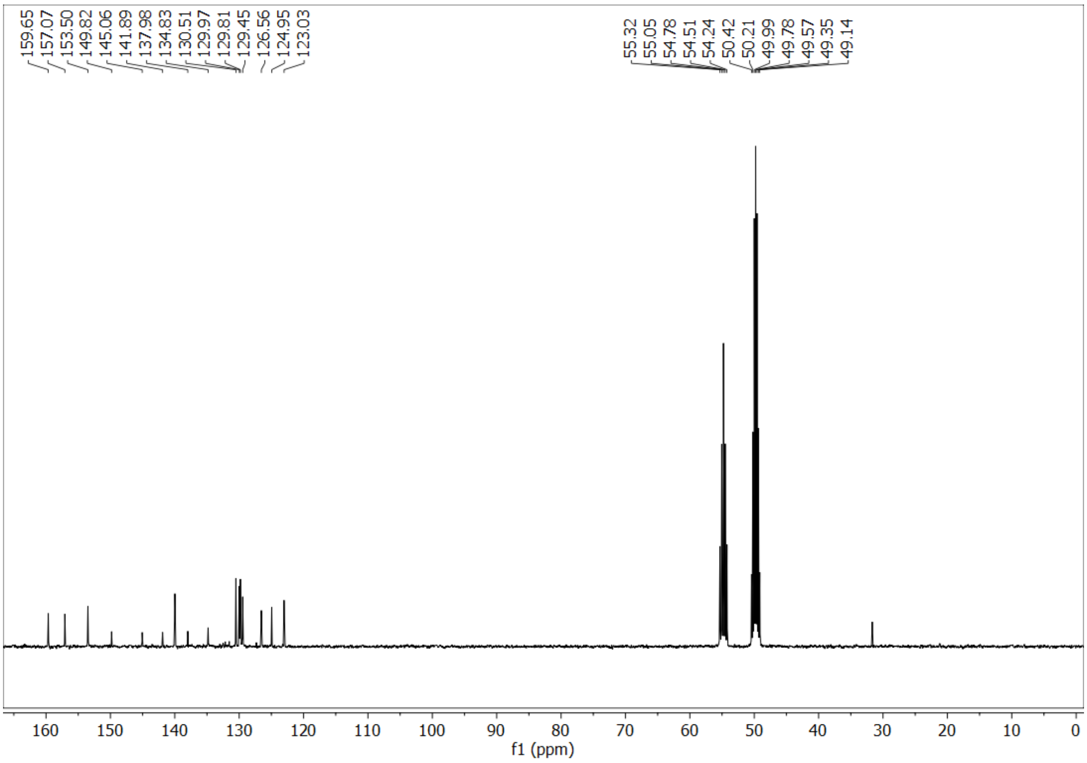

Figure S8. ^{13}C NMR spectrum of 3Ru in CD_2Cl_2/CD_3OD; 1:1 (v/v).

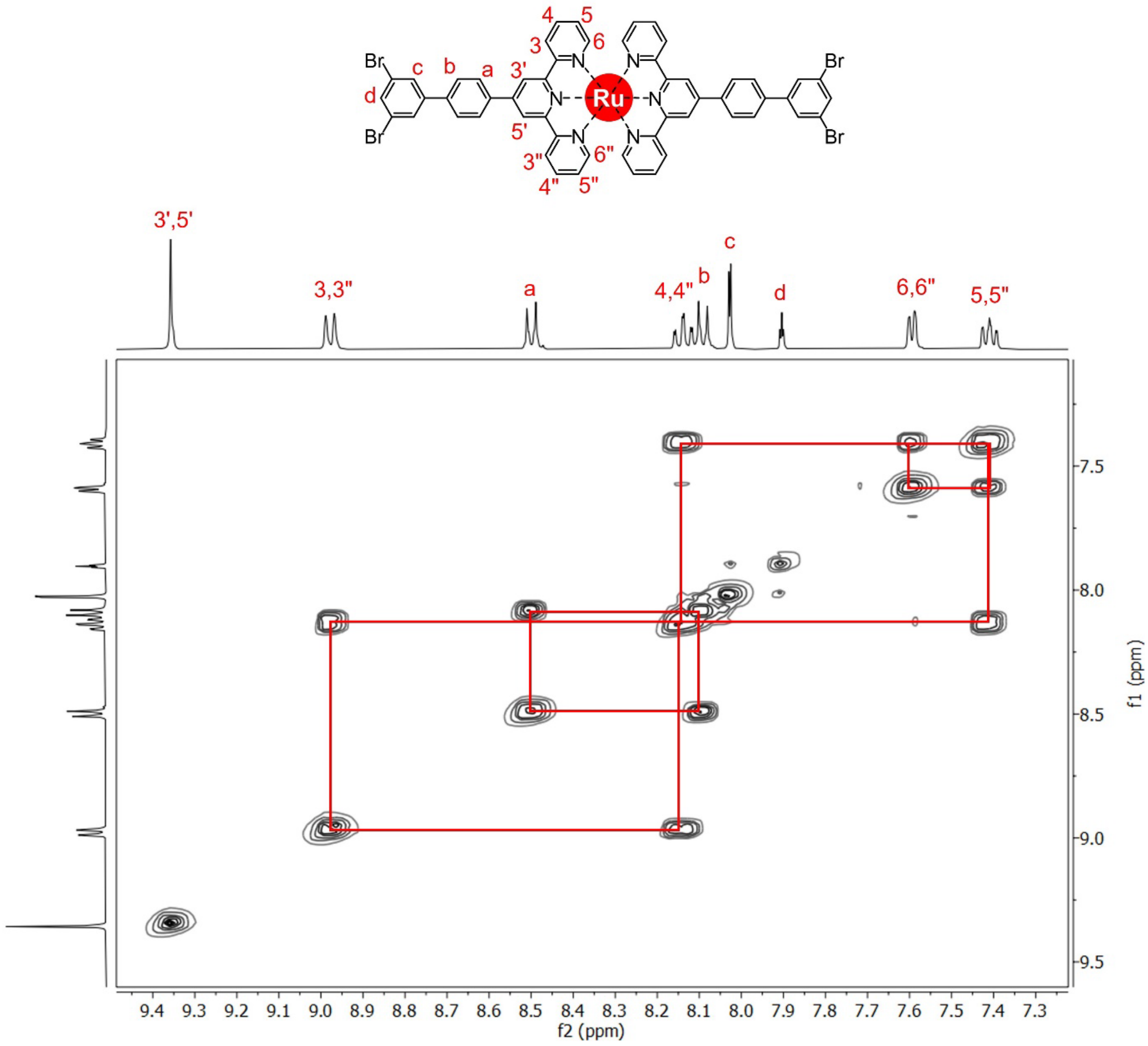

Figure S9. COSY NMR spectrum of 3Ru in CD_2Cl_2/CD_3OD; 1:1 (v/v).

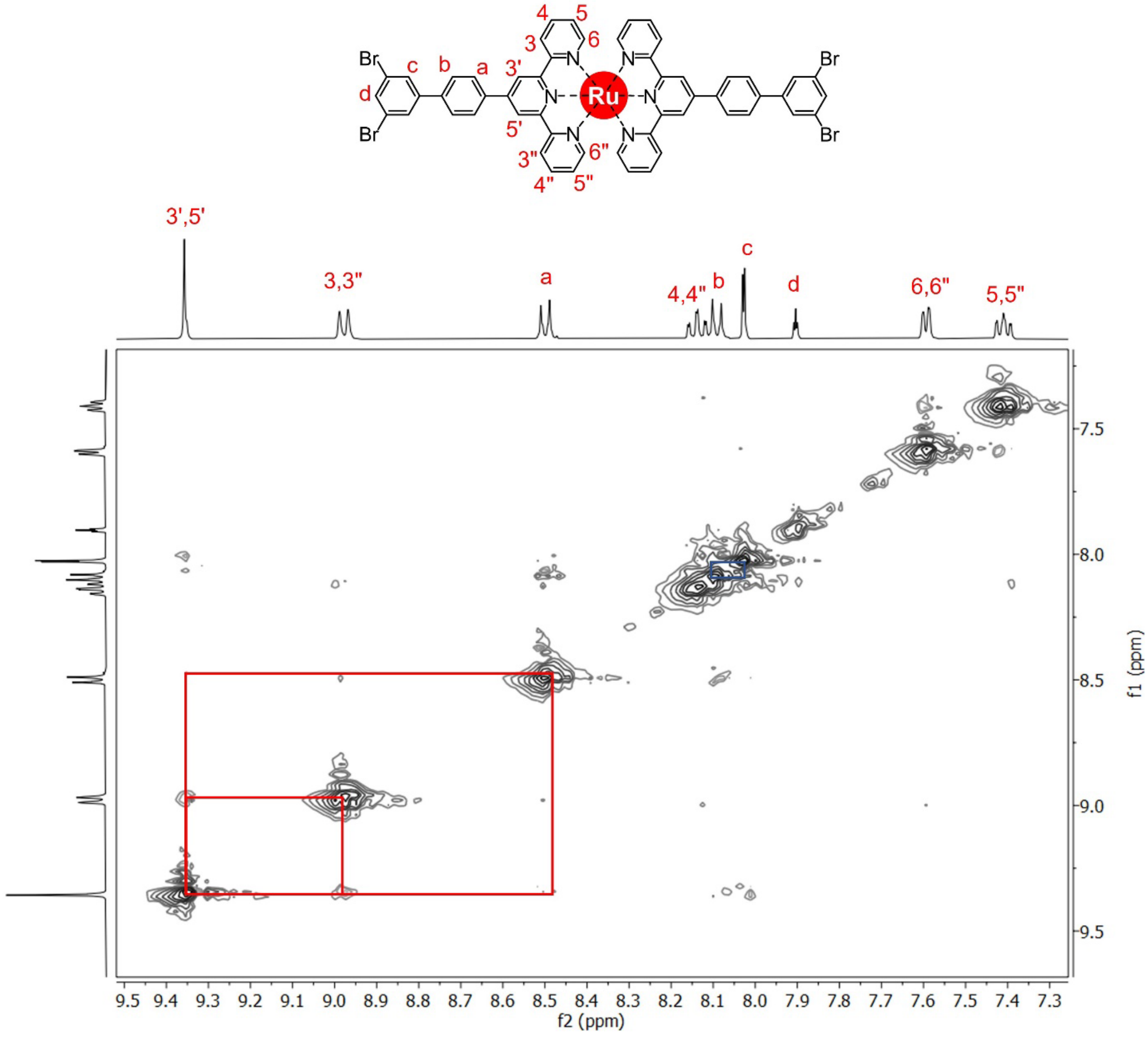

Figure S10. NOESY NMR spectrum of 3Ru in CD_2Cl_2/CD_3OD; 1:1 (v/v).

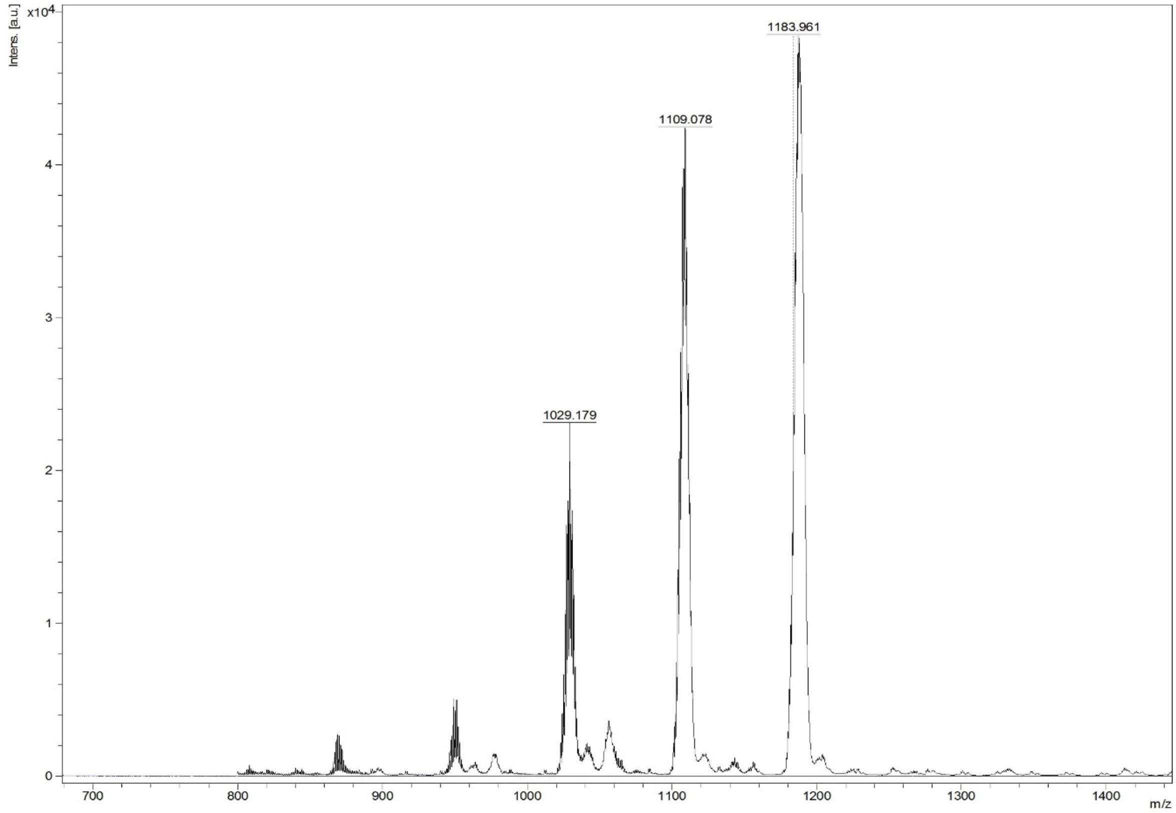

Figure S11. MALDI-TOF mass spectrum of 3Ru.

2.4. Synthesis of 5Os

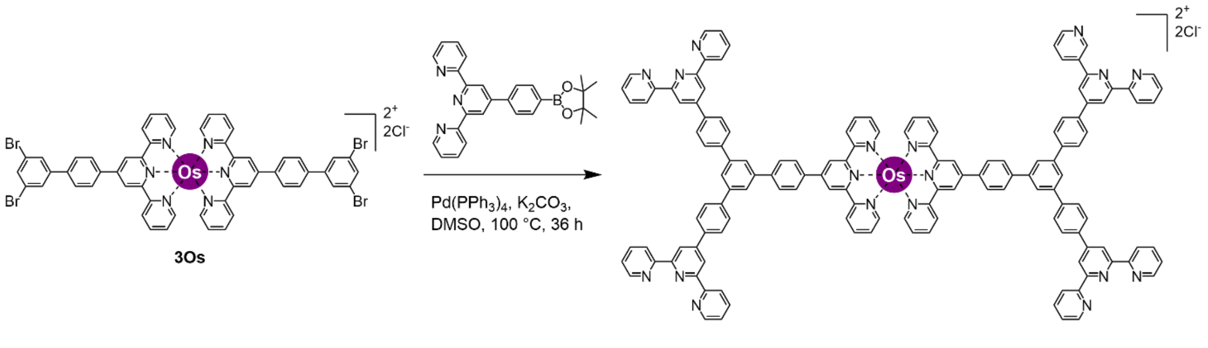

3Os (100 mg, 0.074 mmol), 4'-(4-(4,4,5,5-tetramethyl-1,3,2-dioxaborolan-2-yl)phenyl)-2,2':6',2''-terpyridine (387.6 mg, 0.89 mmol) and K_2CO_3 (25 mg, 0.18 mmol) were dissolved in 15 mL anhydrous DMSO under nitrogen atmosphere. $Pd(PPh_3)_4$ (11.6 mg, 0.011 mmol, 15%) was added to the solution and heated at 100 °C for 36 h. The DMSO was removed under vacuum and resulting solid was purified by column chromatography (basic Al_2O_3) eluting with DCM/MeOH (20:1) to isolate 5Os (113.9 mg, 72 % yield). **^{1}H NMR** (CD_2Cl_2/CD_3OD; 4:1, 400 MHz, ppm): δ 9.34 (s, 4H), 8.98 (s, 8H), 8.90-8.86 (m, 20H), 8.51 (d, 4H), 8.38 (m, 16H), 8.22 (d, 8H), 8.16 (dt, 8H), 8.07 (dd, 4H), 7.63-7.60 (m, 8H), 7.50 (d, 4H), 7.41 (dd, 4H). **^{13}C NMR** (CD_2Cl_2/CD_3OD; 4:1, 400 MHz, ppm): δ 159.15, 155.41, 154.43, 151.35, 149.03, 148.32, 147.26, 141.29, 140.94, 137.62, 137.62, 137.11, 136.79, 127.97, 127.75, 127.57, 127.38, 127.14, 124.75, 124.44, 123.57, 121.09, 119.47, 118.00. **MALDI-TOF-MS** (*m/z*): 2190.11 $[5Os\text{-}2Cl^-]^{2+}$ (calculated *m/z* = 2190.72).

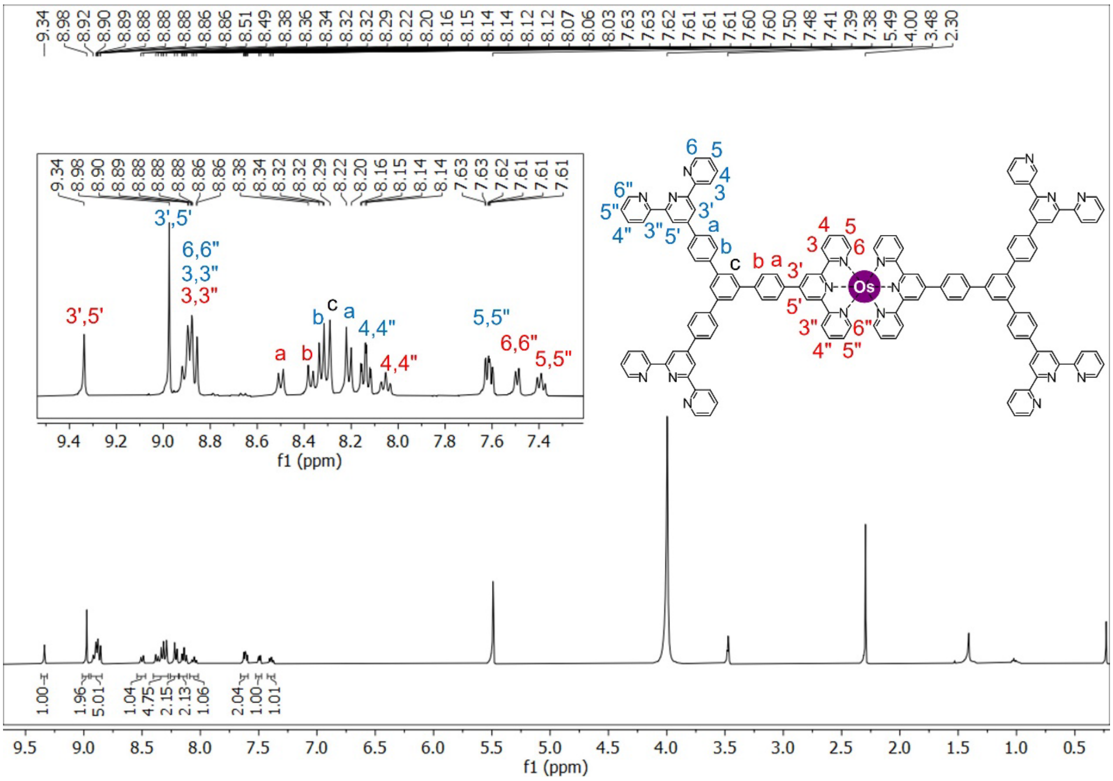

Figure S12. ^{1}H NMR spectrum of 5Os in CD_2Cl_2/CD_3OD; 4:1 (v/v).

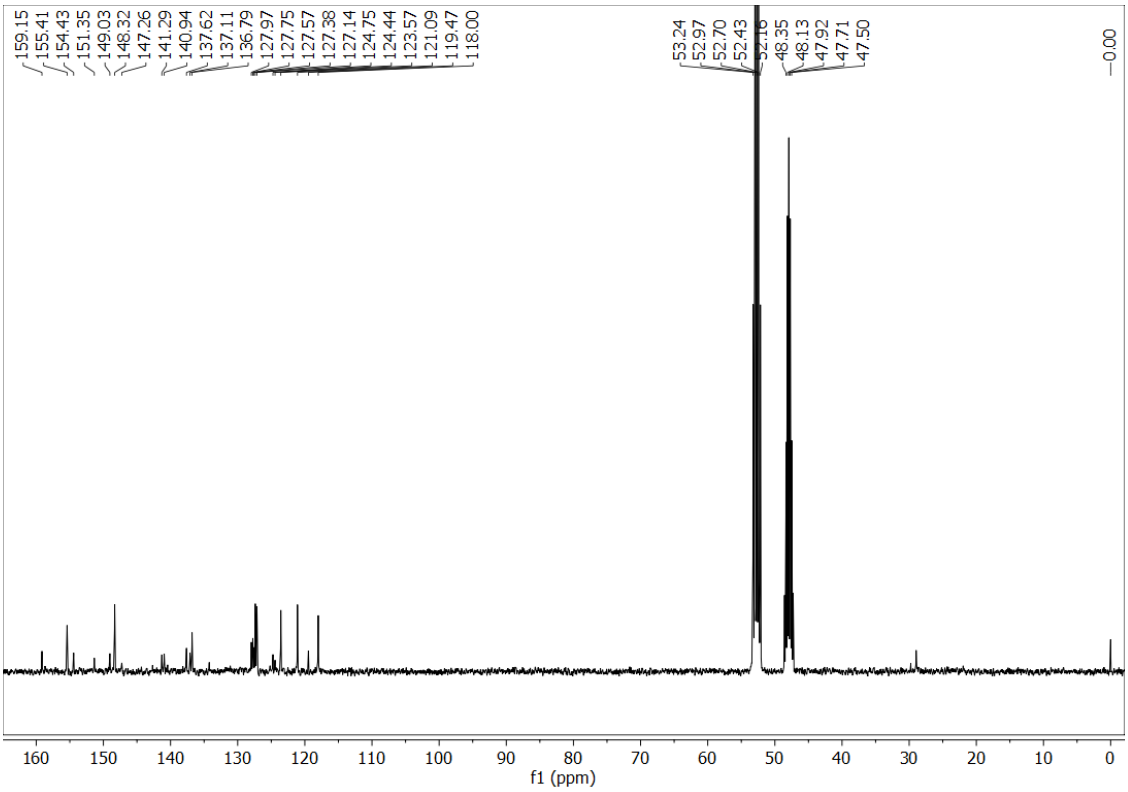

Figure S13. ^{13}C NMR spectrum of 5Os in CD_2Cl_2/CD_3OD; 4:1 (v/v).

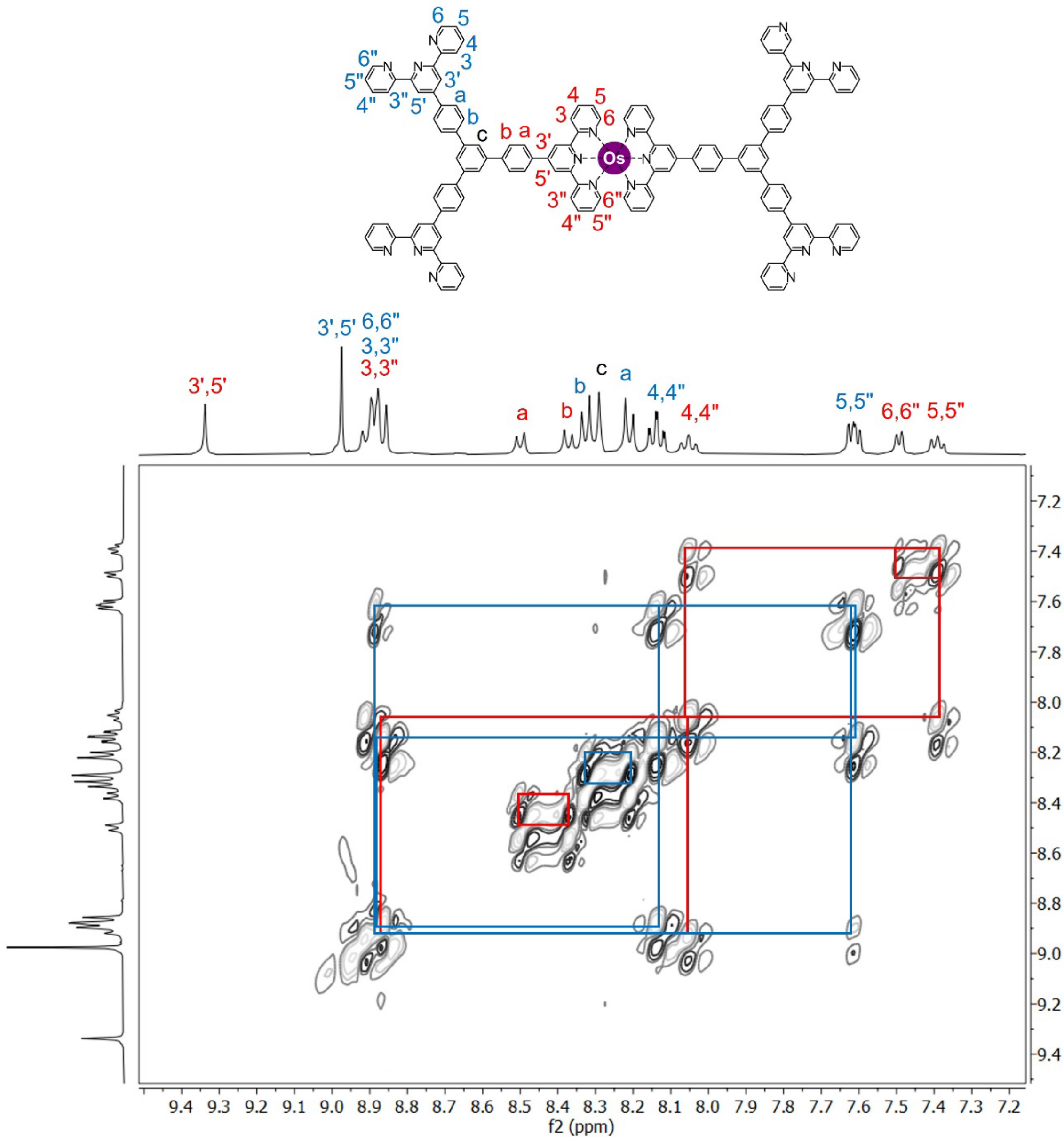

Figure S14. COSY NMR spectrum of 5Os in CD_2Cl_2/CD_3OD; 4:1 (v/v).

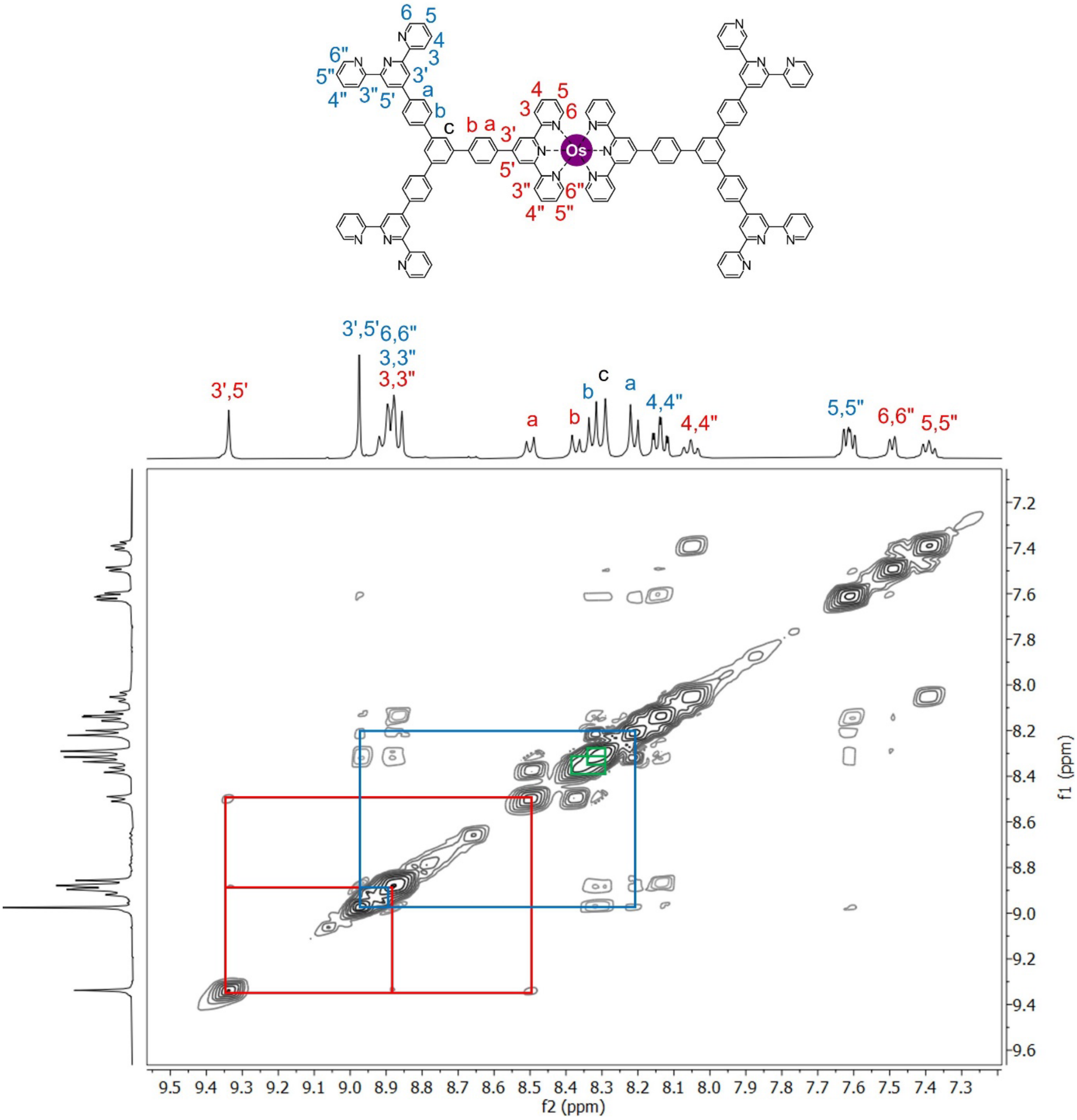

Figure S15. NOESY NMR spectrum of 5Os in CD_2Cl_2/CD_3OD; 4:1 (v/v).

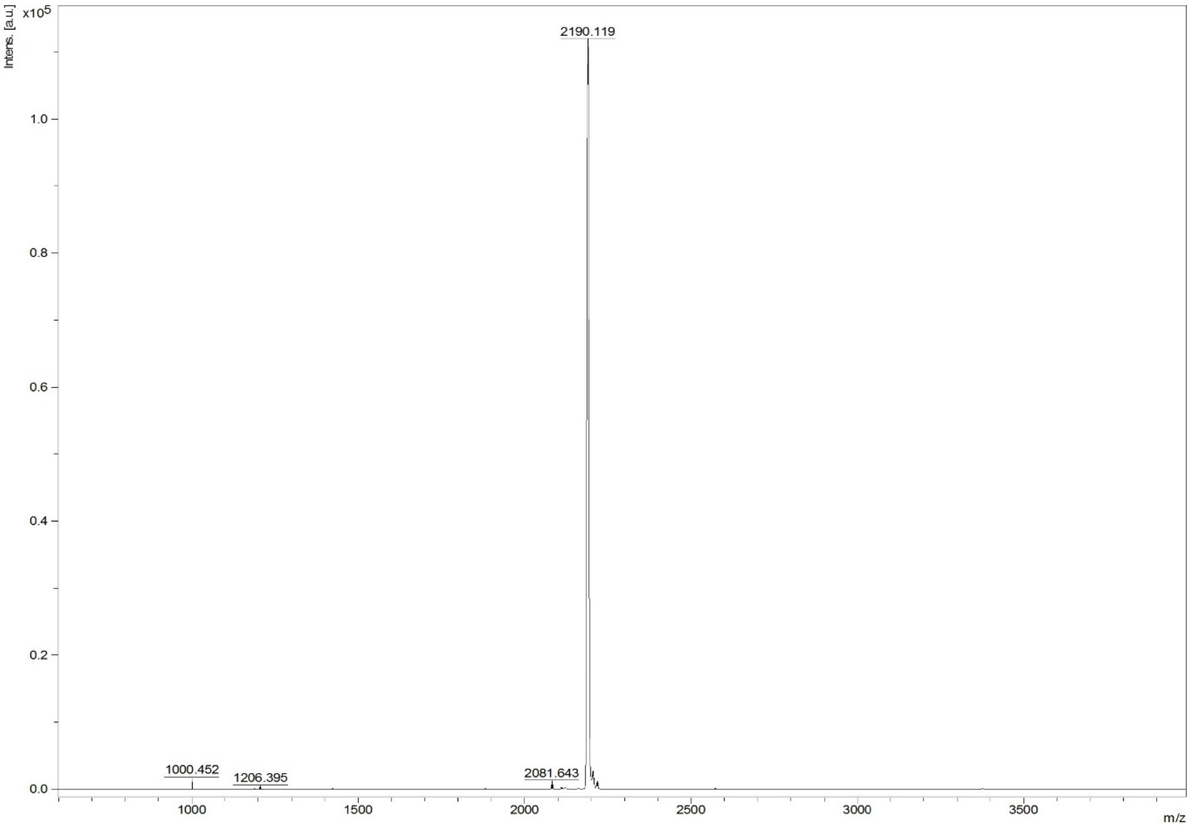

Figure S16. MALDI-TOF mass spectrum of 5Os.

2.5. Synthesis of 5Ru

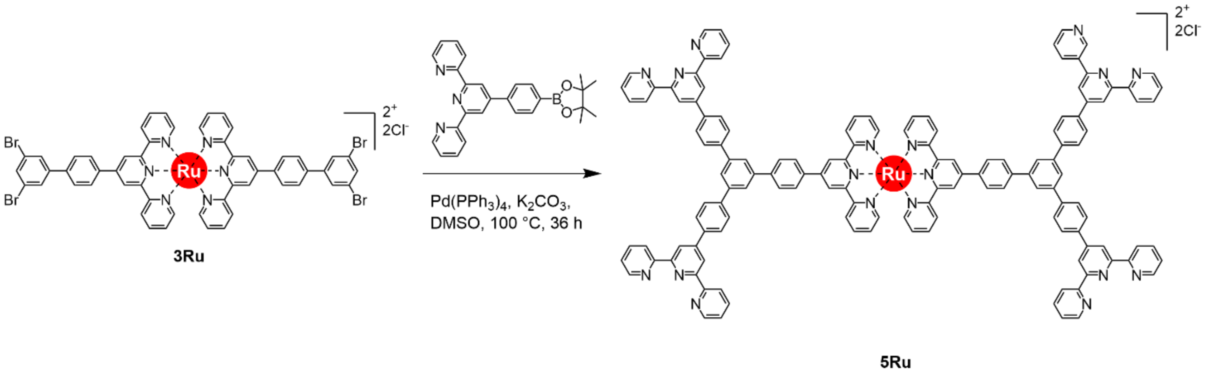

3Ru (147 mg, 0.116 mmol), 4'-(4-(4,4,5,5-tetramethyl-1,3,2-dioxaborolan-2-yl)phenyl)-2,2':6',2''-terpyridine (605.9 mg, 1.39 mmol) and K_2CO_3 (40 mg, 0.29 mmol) were dissolved in 15 mL anhydrous DMSO under nitrogen atmosphere. $Pd(PPh_3)_4$ (19.6 mg, 0.017 mmol, 15%) was added to the solution and heated at 100 °C for 36 h. The DMSO was removed under vacuum and resulting solid was purified by column chromatography (basic Al_2O_3) eluting with DCM/MeOH (20:1) to isolate 5Ru (173.8 mg, 69 % yield). **^{1}H NMR** (CD_2Cl_2/CD_3OD; 4:1, 400 MHz, ppm): δ 9.32 (s, 4H), 8.99 (s, 8H), 8.95 (d, 4H), 8.90-8.86 (m, 16H), 8.56 (d, 4H), 8.39(d, 4H), 8.36 (m, 14H), 8.22 (d, 12H), 8.18-8.11(m, 8H), 7.63(dt, 12H), 7.48(dt, 4H). **^{13}C NMR** (CD_2Cl_2/CD_3OD; 4:1, 400 MHz, ppm): δ 159.53, 157.52, 156.94, 153.44, 151.17, 150.40, 144.77, 143.43, 143.07, 142.62, 140.00, 139.18, 138.98, 130.08, 129.70, 129.49, 129.23, 127.24, 126.77, 126.39, 125.73, 123.29, 122.82, 120.13. **MALDI-TOF-MS** (*m/z*): 2100.94 $[5Ru\text{-}2Cl^-]^{2+}$ (calculated *m/z* = 2100.66).

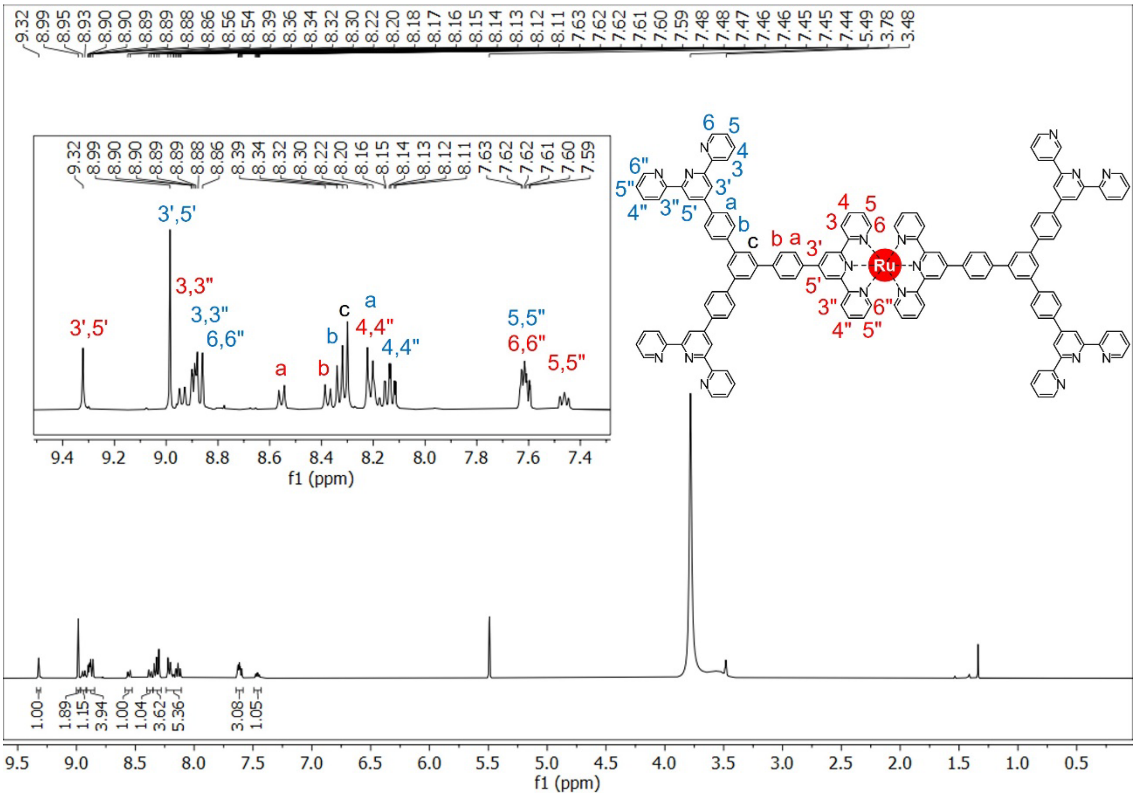

Figure S17. ^{1}H NMR spectrum of 5Ru in CD_2Cl_2/CD_3OD; 4:1 (v/v).

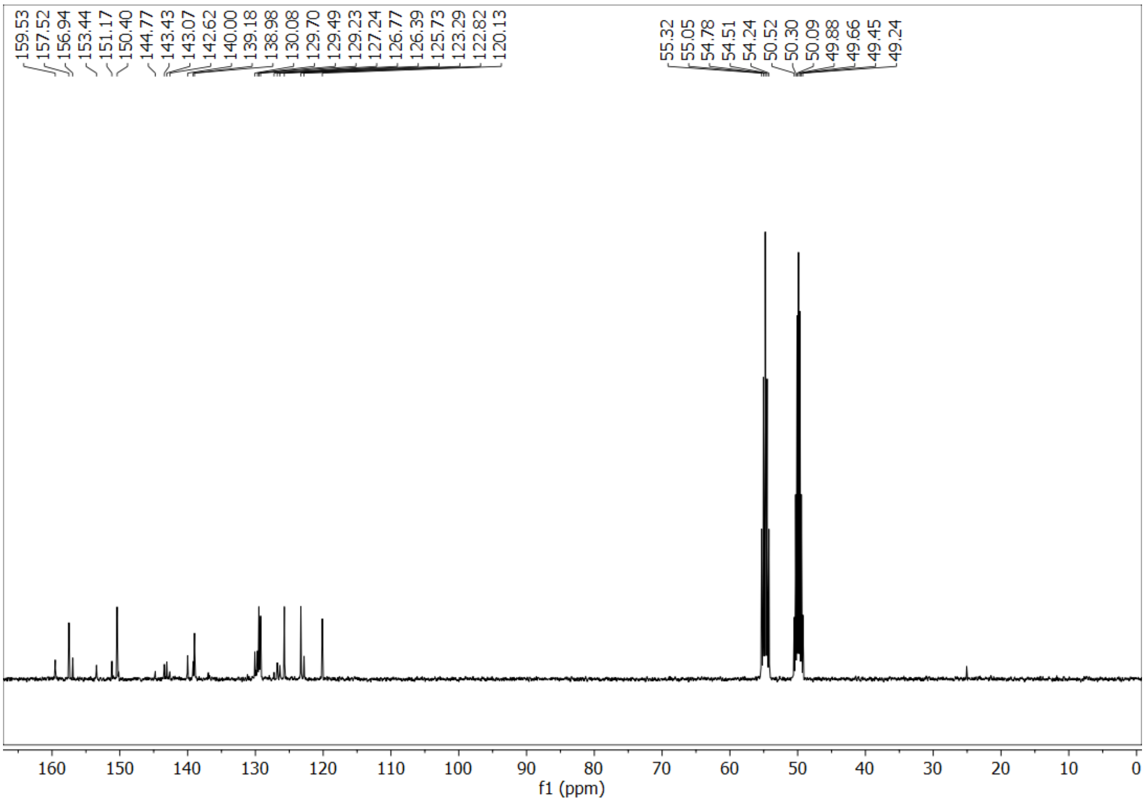

Figure S18. ^{13}C NMR spectrum of 5Ru in CD_2Cl_2/CD_3OD; 4:1 (v/v).

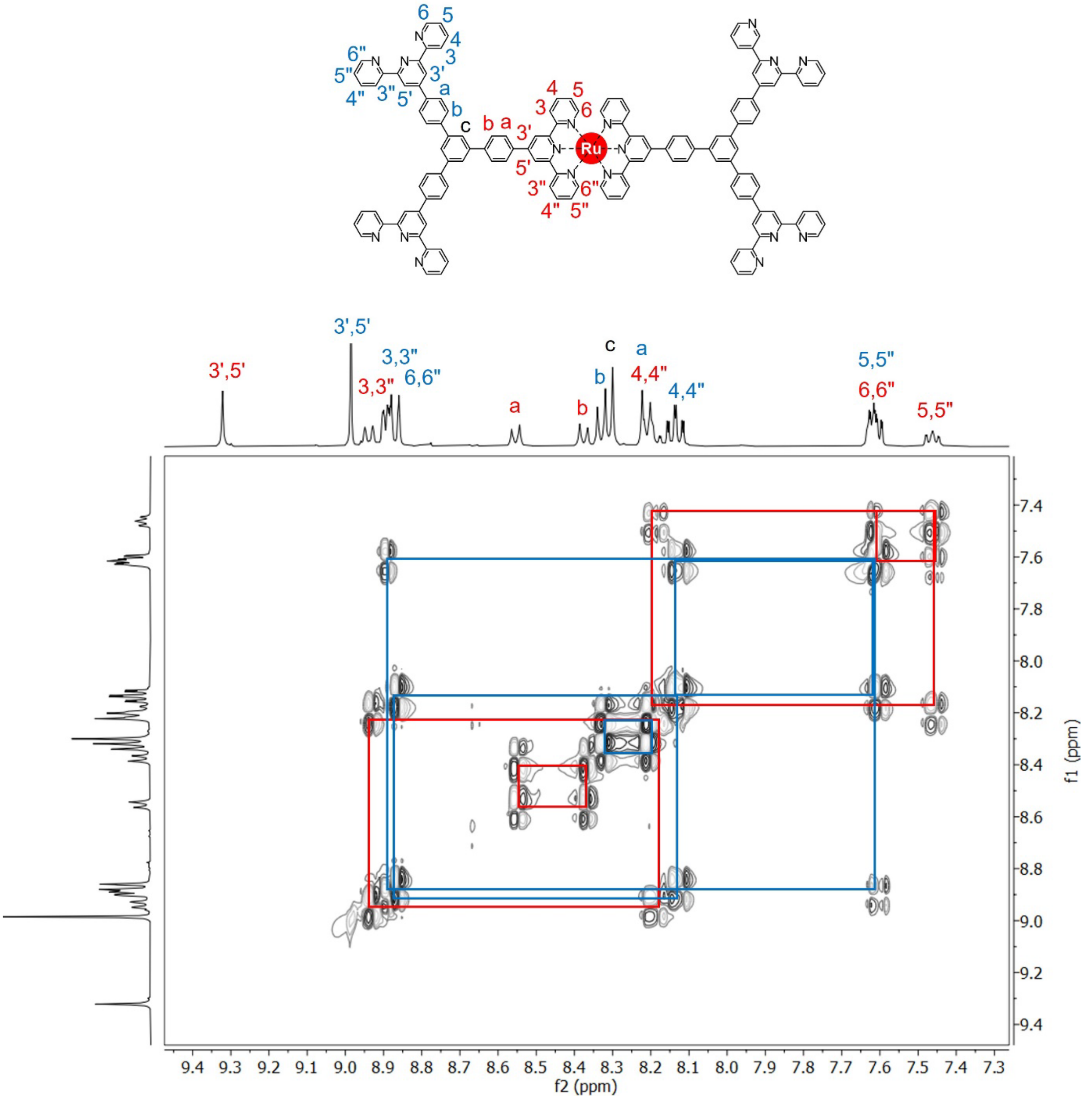

Figure S19. COSY NMR spectrum of 5Ru in CD_2Cl_2/CD_3OD; 4:1 (v/v).

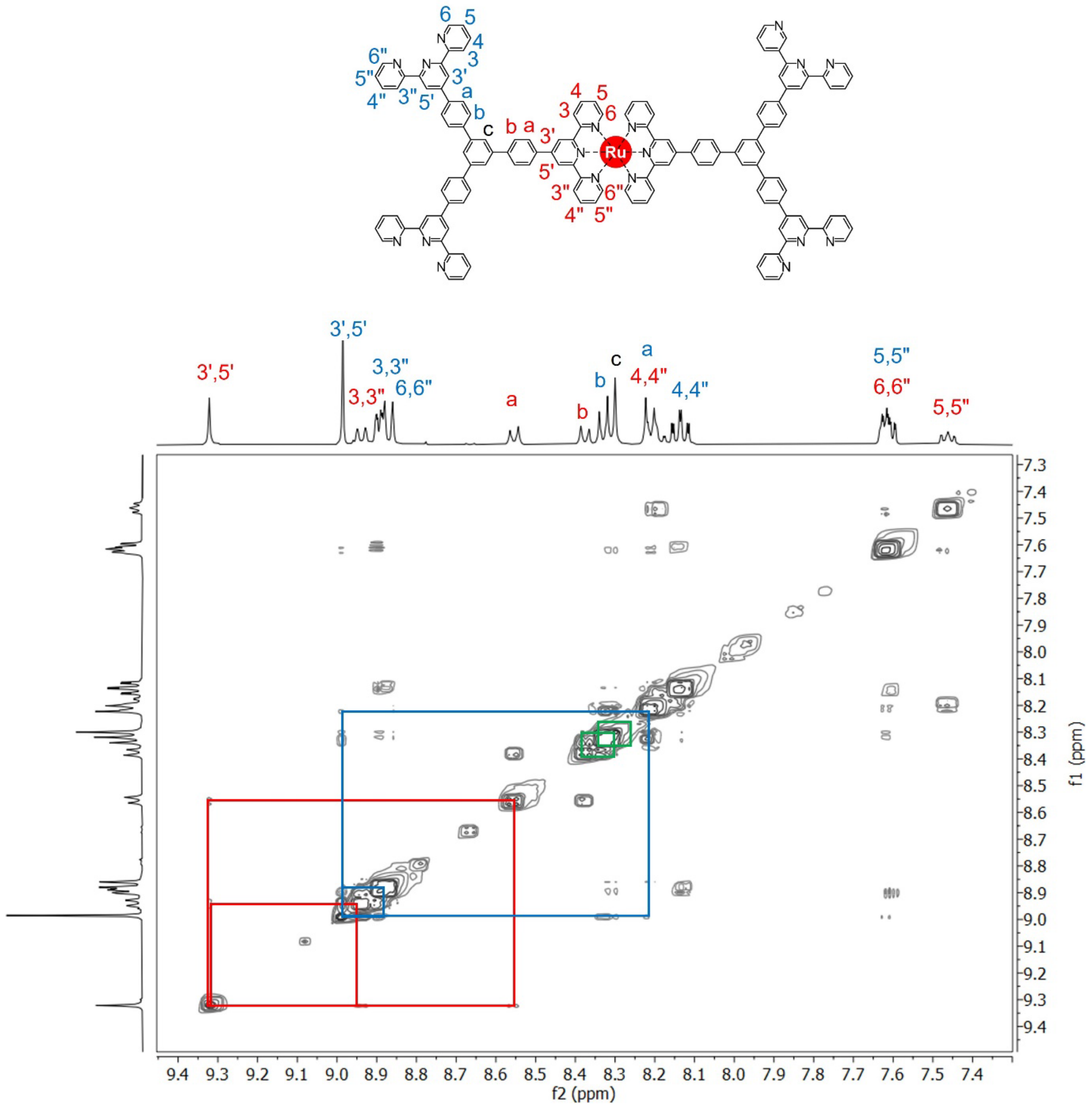

Figure S20. NOESY NMR spectrum of 5Ru in CD_2Cl_2/CD_3OD; 4:1 (v/v).

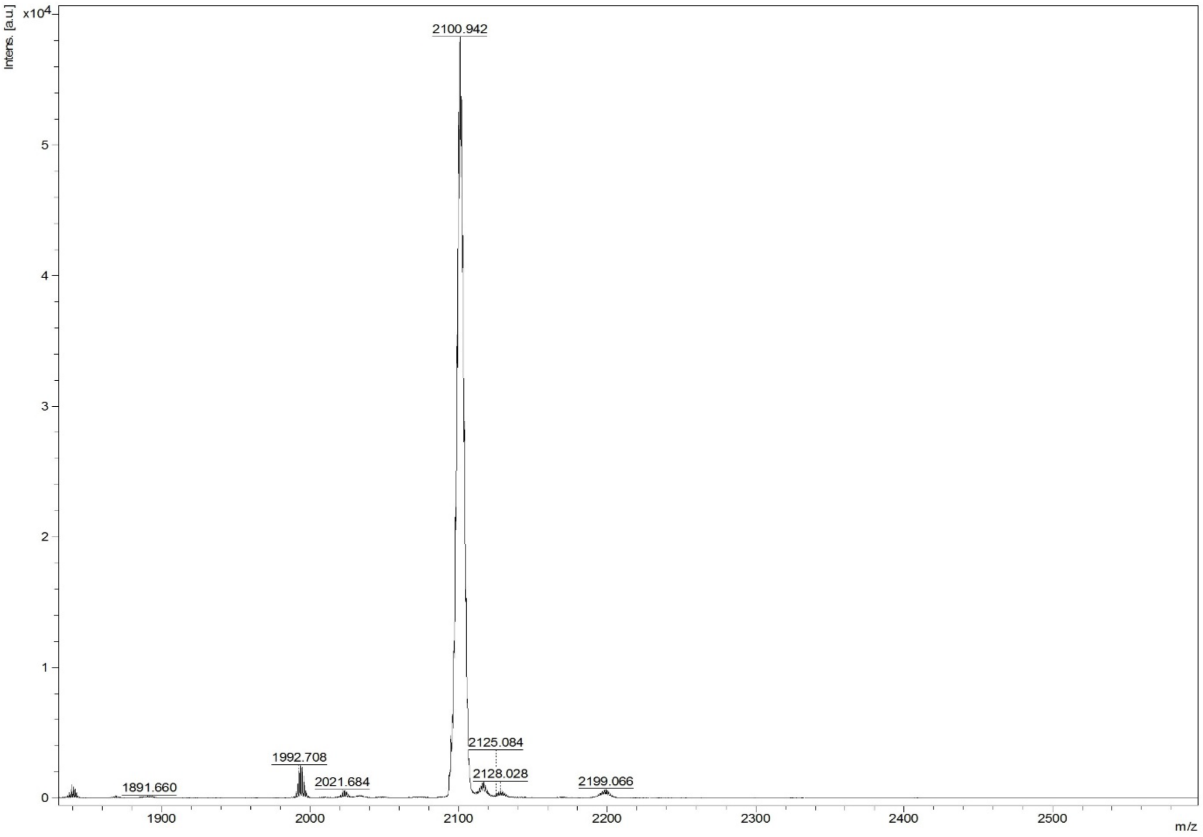

Figure S21. MALDI-TOF mass spectrum of 5Ru.

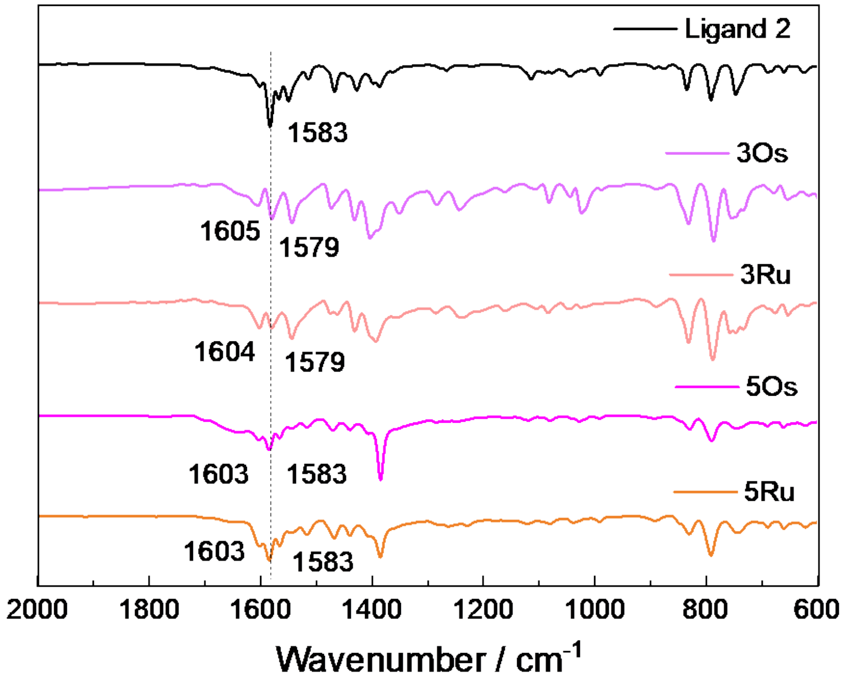

Figure S22. FT-IR spectra of intermediate compounds and metalloligands.

In FTIR spectra of intermediate compounds and metalloligands (Figure S22), the peaks at 1583, 1579, and 1583 cm^{-1}, correspond to C=C stretching frequencies of free terpyridine units, and the peaks at 1605, 1604, and 1603 cm^{-1} correspond to C=C stretching frequencies of complexed terpyridine units.

2.6. Synthesis of heterometallic coordination nanosheets (HMCONASHs)

To prepare HMCONASHs, first the metalloligands (5Os or 5Ru) were dissolved in CH_2Cl_2/CH_3OH (4:1; 1×10^{-4} M) and the solution was filtered using nylon syringe filter (Millipore Millex-HN, 0.45 µm). Taking 10 mL filtered solution of metalloligands in a 100 mL beaker, a liquid-liquid interface was created by gently layering 5 mL double distilled water on the top of organic phase and kept for 30 minutes. Then 5 mL aqueous solution of iron dichloride (filtered before use; 1×10^{-2} M,) was added slowly to aqueous phase. After 8 h, the coloured film of the HMCONASHs (purple for HMCNOsFe and red for HMCNRuFe) was created at the interface. The aqueous phase was exchanged with pure water, followed by the removal of both organic and aqueous phases. A mixture of CH_2Cl_2 and ethanol (1:1) was added to the HMCONASHs film, that made a suspension as flakes. Finally, the flakes were collected by filtration and dried under vacuum for further characterization. The HMCONASHs films were deposited on ITO/glass substrate by keeping the substrate at the bottom of beaker before starting of synthesis. After removal of organic and aqueous phase, the in situ film is deposited on the ITO/glass.

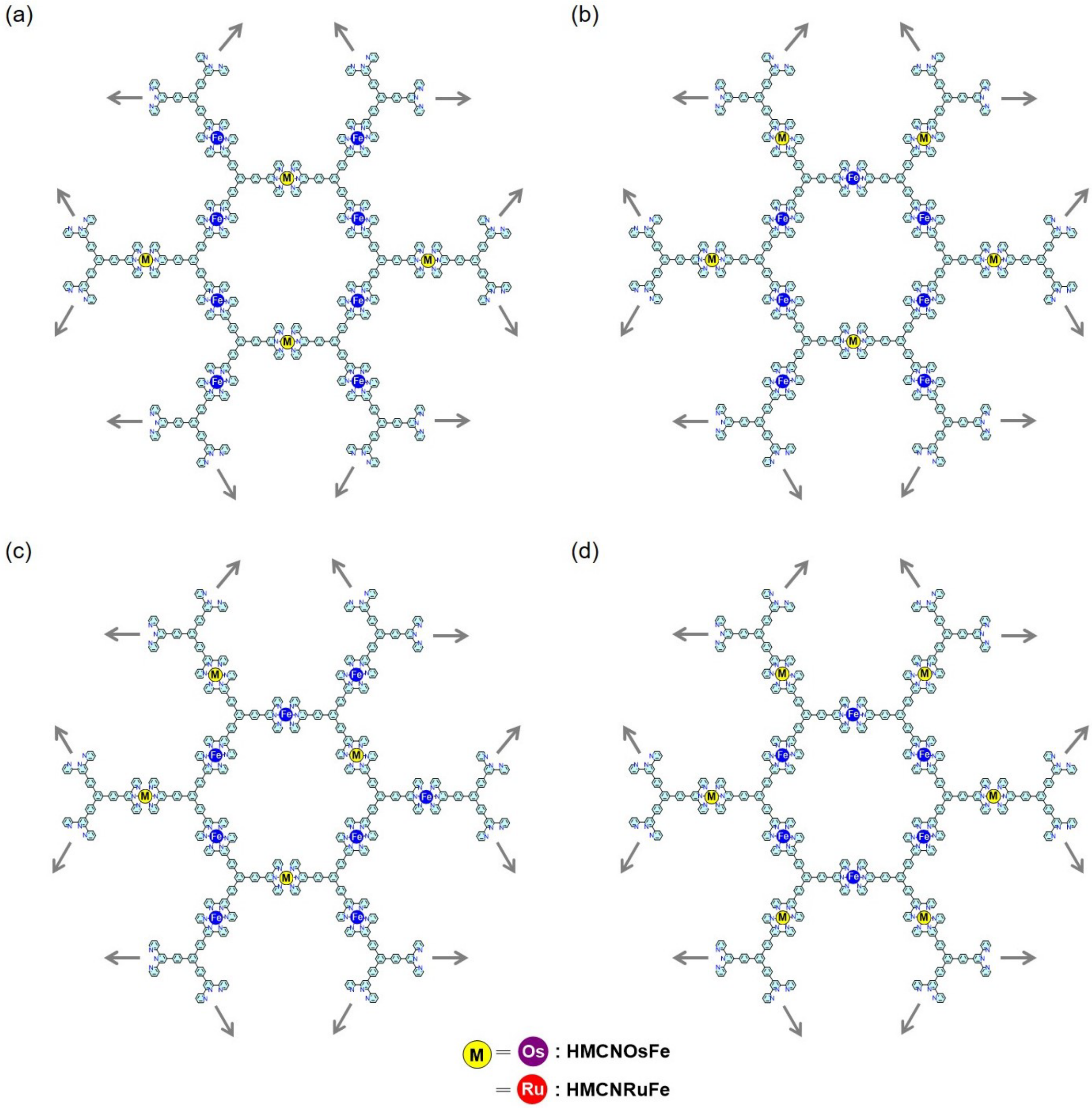

Figure S23. (a-d) Chemical structures of some possible structural isomers for the HMCONSHs (one hexagonal structure is highlighted). Counteranions are omitted for clarity.

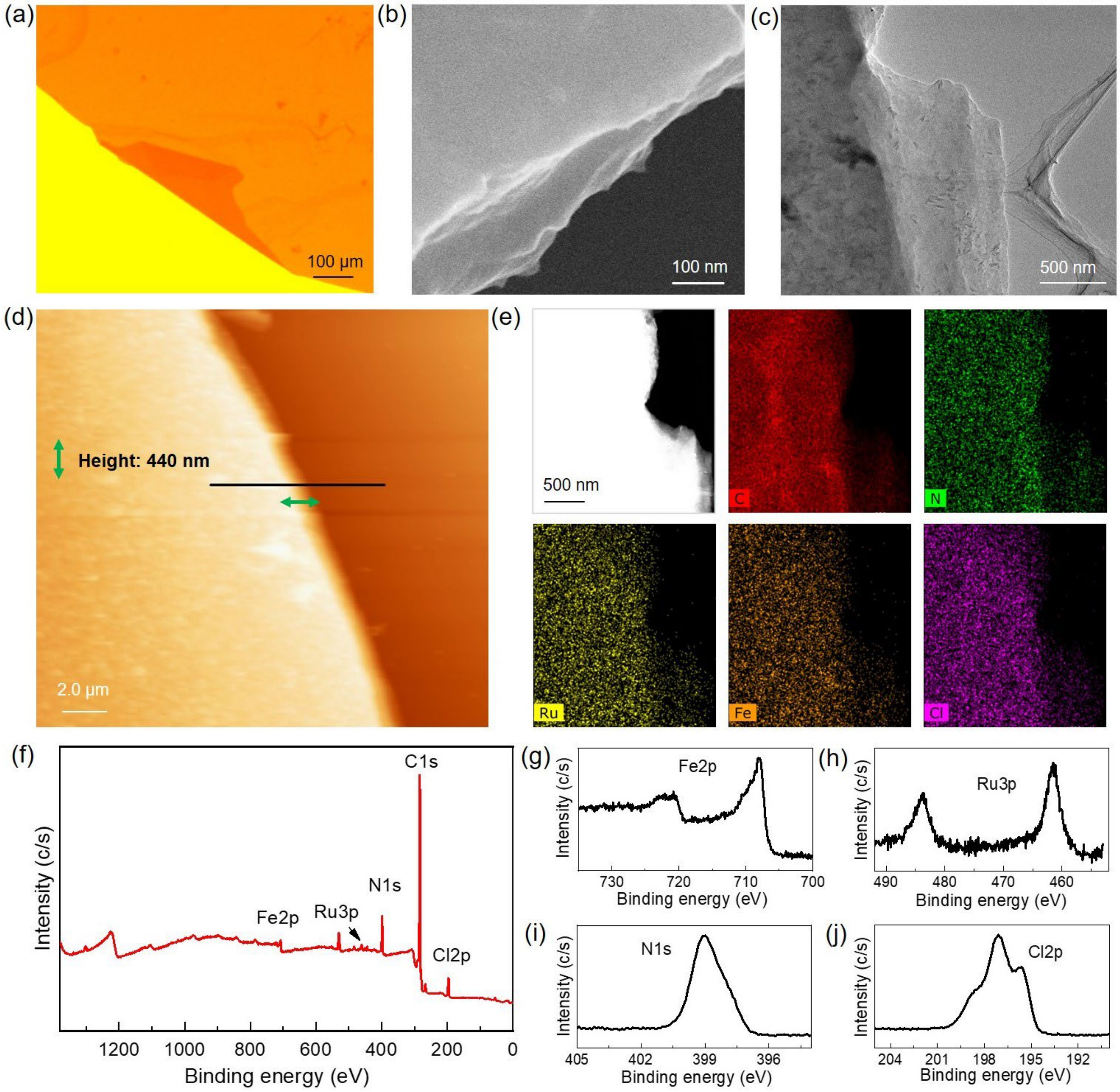

Figure S24. Characterization of HMCNRuFe film. (a) OM, (b) SEM, (c) TEM, (d) AFM image, and (e) TEM/EDX elemental mapping of the film. (f) broad scan and (g-j) narrow scan XP spectra focussing of Ru 3p, Fe 2p, N1s, and Cl 2p core levels, respectively.

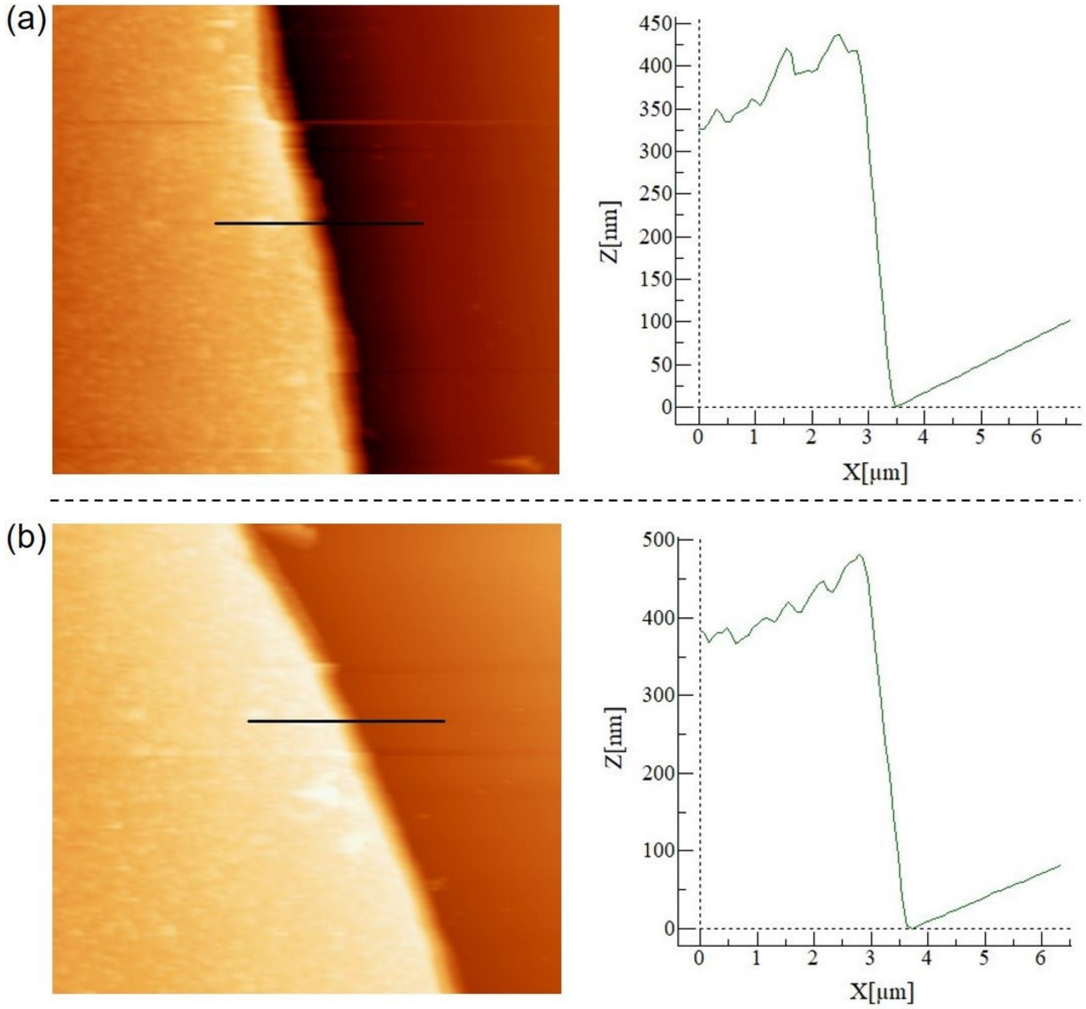

Figure S25. AFM height profile for (a) HMCNOsFe and (b) HMCNRuFe.

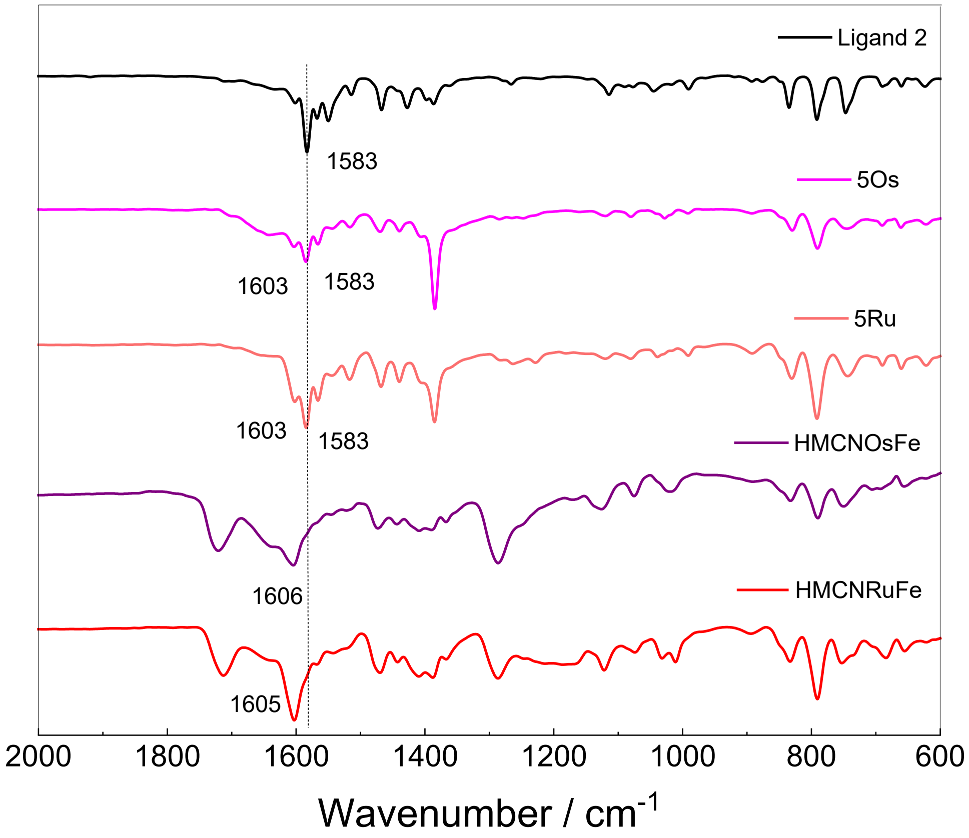

Figure S26. FT-IR spectra of HMCONASHs and comparison with metalloligands.

In the FTIR spectra (Figure S26), the peaks at 1605, and 1606 cm^{-1}, correspond to C=C stretching frequencies of complexed terpyridine units of the HMCONASHs. Compared to metalloligands, the HMCONASHs show only C=C stretching frequencies of complexed terpyridine units.

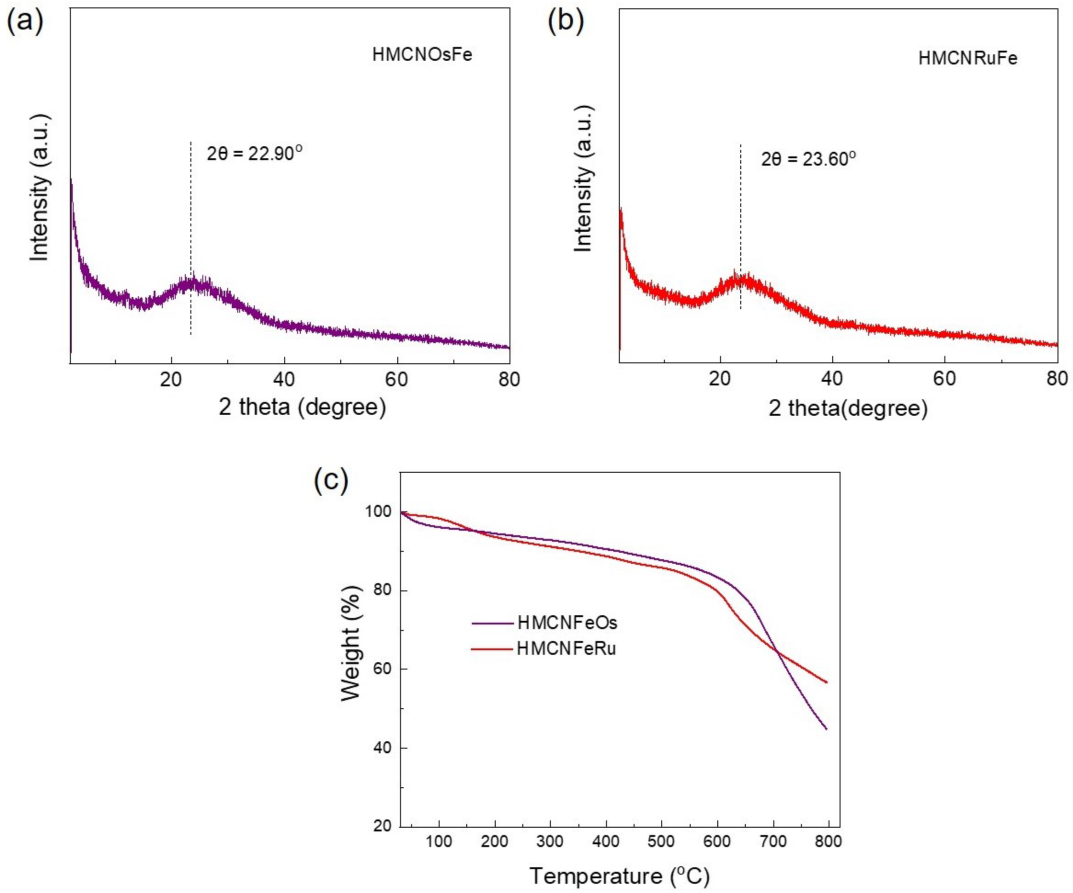

Figure S27. (a-b) The PXRD patterns of HMCONASHs. (c) Thermal gravimetric analysis (TGA) profile for HMCONASHs.

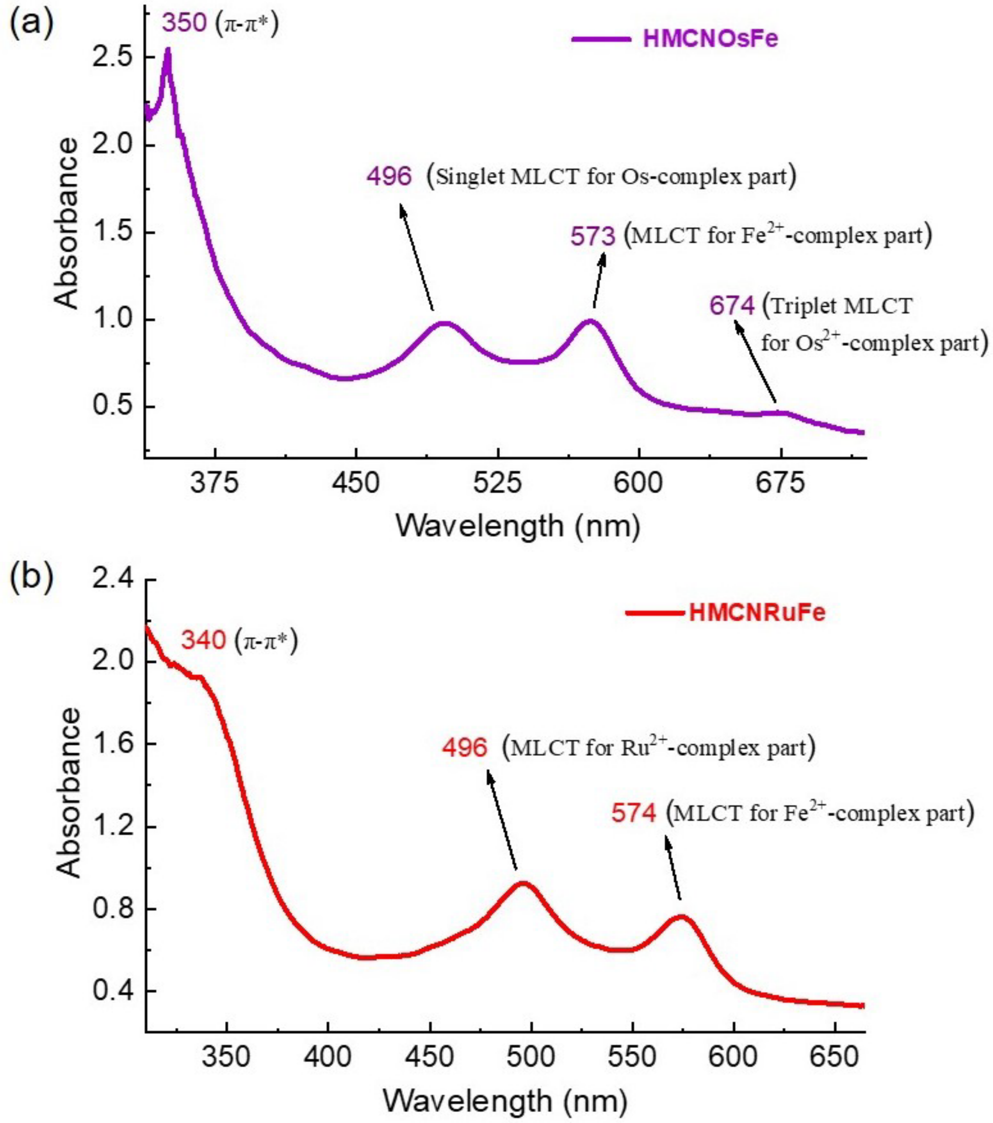

Figure S28. UV-vis spectra of HMCONASHs film for (a) HMCNOsFe and (b) HMCNRuFe with assignment of different peaks.

Table ST1. Comparison of our work with the earlier reports.

Ligand structure	Network Structure for coordination nanosheets	Weak coordination metal ion	Strong coordination metal ion	Type of nanosheets	References
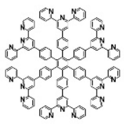	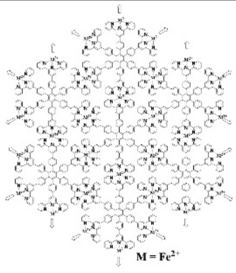	Fe^{2+}	-	Homometallic CONASHs	*Angew. Chem. Int. Ed.* **2011**, 50 (34), 7879-7884
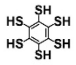	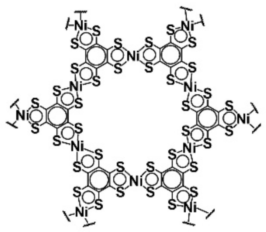	Ni^{2+}	-	Homometallic CONASHs	*J. Am. Chem. Soc.* **2013**, 135 (7), 2462-2465
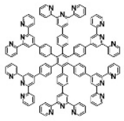	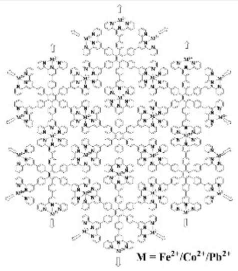	Fe^{2+} / Co^{2+} / Pb^{2+} (Via transmetallation of Zn^{2+} nanosheets)	-	Homometallic CONASHs	*J. Am. Chem. Soc.* **2014**, *136* (16), 6103-6110
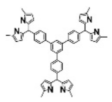	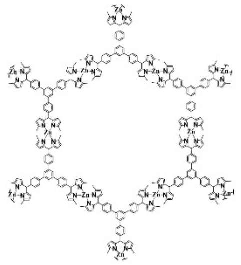	Zn^{2+}	-	Homometallic CONASHs	*Nat. Commun.* **2015**, 6 (1), 6713
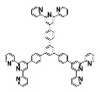	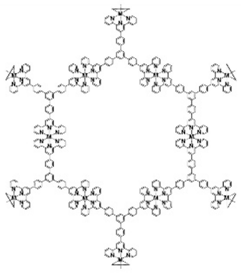	Fe^{2+}, Co^{2+}	-	Homometallic CONASHs	*J. Am. Chem. Soc.* **2015**, *137* (14), 4681-4689

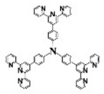	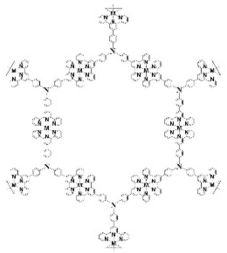	Zn^{2+}	-	Homometallic CONASHs	*J. Am. Chem. Soc.* **2017**, *139* (15), 5359-5366
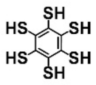	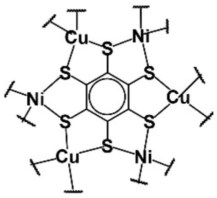	Cu^{2+} & Ni^{2+}	-	Heterometallic CONASHs	*Adv. Mater* **2022**, 34 (13), 2106204
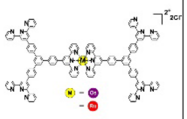	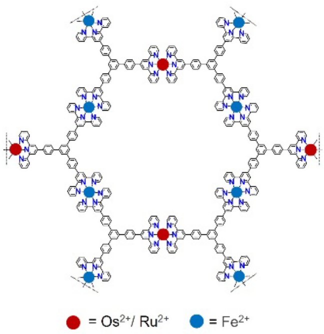	**Fe^{2+}**	**Os^{2+} / Ru^{2+}**	• The First heterometallic CONASHs with both strong & weak coordination metal ions together<br>• Showing dual redox activity<br>• Showing multicolor electrodhromism	**This work**

References:

1. Capitosti, G. J.; Guerrero, C. D.; Binkley, D. E.; Rajesh, C. S.; Modarelli, D. A., Efficient Synthesis of Porphyrin-Containing, Benzoquinone-Terminated, Rigid Polyphenylene Dendrimers. *J. Org. Chem.* **2003,** *68* (2), 247-261.

2. Hanwell, M. D.; Curtis, D. E.; Lonie, D. C.; Vandermeersch, T.; Zurek, E.; Hutchison, G. R., Avogadro: an advanced semantic chemical editor, visualization, and analysis platform. *J. Cheminform.* **2012,** *4* (1), 17.

3. Fletcher, R.; Reeves, C. M., Function minimization by conjugate gradients. *Comput. J.* **1964,** *7* (2), 149-154.

4. Rappe, A. K.; Casewit, C. J.; Colwell, K. S.; Goddard, W. A., III; Skiff, W. M., UFF, a full periodic table force field for molecular mechanics and molecular dynamics simulations. *J. Am. Chem. Soc.* **1992,** *114* (25), 10024-10035.
